# Circular dichroism of relativistically–moving chiral molecules

**DOI:** 10.1038/s41598-024-66443-w

**Published:** 2024-07-22

**Authors:** Mitchell R. Whittam, Benedikt Zerulla, Marjan Krstić, Maxim Vavilin, Christof Holzer, Markus Nyman, Lukas Rebholz, Ivan Fernandez-Corbaton, Carsten Rockstuhl

**Affiliations:** 1 Institute of Theoretical Solid State Physics, Karlsruhe Institute of Technology (KIT), Kaiserstr. 12, 76131 Karlsruhe, Germany; 2 Institute of Nanotechnology, Karlsruhe Institute of Technology (KIT), Kaiserstr. 12, 76131 Karlsruhe, Germany

**Keywords:** Chiral molecules, Relativistic motion, Circular dichroism, Multi–scale modelling, Quantum chemistry, Nanophotonics and plasmonics, Chemical physics

## Abstract

Understanding the impact of the relativistic motion of a chiral molecule on its optical response is a prime challenge for fundamental science, but it also has a direct practical relevance in our search for extraterrestrial life. To contribute to these significant developments, we describe a multi–scale computational framework that combines quantum chemistry calculations and full–wave optical simulations to predict the chiral optical response from molecules moving at relativistic speeds. Specifically, the effect of a relativistic motion on the transmission circular dichroism (TCD) of three life–essential biomolecules, namely, B–DNA, chlorophyll *a*, and chlorophyll *b*, is investigated. Inspired by previous experiments to detect interstellar chiral molecules, we assume that the molecules move between a stationary observer and a light source, and we study the rotationally averaged TCD as a function of the speed of the molecule.We find that the TCD spectrum that contains the signatures of the molecules shifts with increasing speed to shorter wavelengths, with the effects already being visible for moderate velocities.

## Introduction

Chirality, a term coined by Lord Kelvin for objects that cannot be superimposed onto their mirror image, continues to be a prime research theme both in general and specifically in the context of nanomaterials. Whereas it might be easy to judge whether a macroscopic object is chiral, it becomes notoriously complicated to probe a possible chirality at the nanoscale. Most of the physical properties of the two enantiomers of a chiral molecule are the same, and to judge whether an object on the nanoscale is chiral, the chirality needs to be probed by another chiral object, i.e., circularly polarised light.

A prime quantity to look at in the scenario of light–matter interaction is the difference in absorption between left– and right–handed circularly polarised light, known as circular dichroism (CD)^1–8^. The CD is a powerful tool in identifying particular molecules and their substructures. For example, one can obtain valuable information about the secondary structures of amino acids and proteins^9^. Moreover, since most biological molecules are chiral^10^, investigating the CD of such molecules helps us to identify particular lifeforms, including extraterrestrial ones^11^. With the recent discovery of the first–known interstellar chiral molecule propylene oxide (CH$_{3}$CHCH$_{2}$O)^12^, studying the chiral response of molecules in such a setting has become a timely issue. In the long run, it can be motivated by our desire to detect other forms of life. Still, it remains also a fundamental scientific challenge to understand the chiral signatures of molecules moving at relativistic speeds.

To contribute to these developments, we investigate the optical response of three life–essential chiral biomolecules, specifically B–DNA, chlorophyll *a*, and chlorophyll *b*. These biomolecules are chosen due to their uniqueness and because they are signature molecules in life forms on Earth. To build upon previous work, we extend this to the relativistic regime, i.e., a regime where the molecules move at a considerable fraction of the speed of light. The idea behind this is to look into the effect of speed on the chiral signature of the biomolecules mentioned above and to see which features of the CD are affected by these high speeds. To our knowledge, such a study has not been published until now, especially one connecting precise quantum chemistry calculations of stationary biomolecules to the scattering simulations of relativistically moving objects. Generally, studies on the scattering of light by relativistically moving objects have been restricted to rather basic shapes, such as spheres^13–15^, and many optical properties, such as the CD we look at here, have not been considered.

To computationally explore the optical signatures from molecules, we exploit a multi–scale modelling framework^16–18^. Firstly, we study the properties of the molecules using density functional theory (DFT). By using time–dependent DFT, we obtain the spectrally resolved bianisotropic polarisabilities of the considered molecules in the dipole approximation. From this analysis, we obtain the transition matrices (called, for brevity, *T*–matrices) for each molecule. The *T*–matrix is a comprehensive representation of the optical properties of an object, in our case, different biomolecules. Once the *T*–matrix is known, we use it to obtain the scattered field, from which the outgoing field is determined.

In our contribution, we go one step further and consider the interaction of light with molecules that are moving at relativistic speeds. To achieve this, we use recent results that allow one to boost generic polychromatic fields^19,20^. Given the plans to create structures that move at relativistic speeds, like the light sail proposed by the Breakthrough Starshot Initiative^21,22^, it is reasonable to assume there could exist biomolecules moving at such speeds. Also, with the increasing interest in measuring chiral signatures of molecules from deep space, it remains an open question what the possible impact of a motion at relativistic speeds would have on observable spectroscopic quantities.

The specific quantity we explore is the Lorentz–boosted rotationally–averaged transmission circular dichroism (TCD) as a function of the speed of the molecule. The TCD is the difference between the transmissions of incoming waves of positive and negative helicity. We assume that the molecules are situated between the light source and the observer, and are moving towards the observer in the $-z$–direction. This is similar to the setup used in Ref. ^12^, where the light source was the giant molecular gas cloud Sagittarius B2 (Sgr B2).

To obtain a rotational average of the TCD signal, we illuminate the molecules in the simulations at multiple angles of orientation by a plane wave and boost the TCD from the molecules’ frame to the lab frame. We consider the rotationally–averaged TCD because the molecules will likely exist in aggregates within which each molecule has a random orientation. Moreover, DNA is built from nucleotide base pairs that have an angular offset to the upper and lower pair along the DNA double–strand, contributing to the rotationally averaged CD signal.

The structure of the paper is as follows. Firstly, we outline the methods to compute the *T*–matrices for each molecule and highlight the considered parameters. Secondly, we discuss the procedure required to determine the Lorentz–boosted TCD. In essence, we rely on results in^19,20^ to implement a frame–hopping formalism. To elaborate on that in detail, we outline the necessary mathematical methods and derivations to boost the incident electric field to the reference frame of the molecules, followed by those to determine the outgoing field in this frame and, consequently, the rotationally–averaged TCD in the lab (observer’s) reference frame. Afterwards, we provide our results showing how the TCD varies in the lab frame with the speed of each molecule and wavelength of the incoming field and discuss them. Finally, we conclude our findings.

## Methods

### Density functional theory calculations for molecules

Quantum chemistry calculations of the finite–size B–DNA molecular model and the chlorophyll *a* and *b* molecules surrounded by water have been performed using DFT and its linear response as implemented in a development version of the TURBOMOLE 7.8 electronic structure program^23,24^. For all three molecules, a Gaussian–type def2–TZVP basis set^25,26^ of triple–$\zeta$–quality with additional valence and polarisation functions was used to describe the orbitals. Water as the surrounding medium that influences the structure and optical properties of the considered molecules was accounted for implicitly through a Conductor–like Screening Model (COSMO). Since the DNA molecule is rather stable in the aqueous surrounding^27,28^, we took the structure from a crystallographic database and used it without re–optimisation in the consequent quantum chemistry calculations. In contrast to DNA, the molecular geometries of chlorophyll *a* and *b* were obtained via an energy minimisation technique. The geometries were subsequently used to calculate the damped complex dynamic polarisability tensors, which account for electric–electric, electric–magnetic, and magnetic–magnetic interactions. In the calculations of the dynamic polarisability tensors of the DNA, the damping parameter was set to 0.15 eV at the half–width at half–maximum (HWHM). In contrast, the same parameter for the chlorophyll molecules was set to 0.03 eV. The different damping was chosen for these biomolecules due to the different spectral windows where optical processes were simulated.

The finite–size molecular model of DNA was composed of eight nucleotide base pairs. The hybrid PBE0 functional^29,30^ was used in the density functional calculations. Additionally, two structurally different chlorophyll molecules denoted *a* and *b* with distinctive linear absorption properties were considered. In contrast to the DNA molecule, we used for chlorophyll *a* hybrid TPSSh DFT functional^31–33^ to account for the positions of low–lying electronic transitions in the absorption spectrum.

Additionally, our simulations made extensive use of the resolution–of–identity (RI) approximation^34,35^, together with the multipole accelerated resolution–of–identity (marij) approximation^36^, and seminumerical calculation of exchange (senex, esenex) algorithms^37^ to speed up the simulations significantly without influence on the quality of the results.

Finally, dynamic polarisabilities obtained from quantum chemistry calculations allowed us to construct *T*–matrices for these molecules^17,38^. We use the following relation:

1 $$\begin{aligned} \begin{aligned} {\textbf{T}}= \begin{pmatrix} {{\textbf{T}}_{\textrm{NN}}}&{{\textbf{T}}_{\textrm{NM}}}\\ {{\textbf{T}}_{\textrm{MN}}}&{{\textbf{T}}_{\textrm{MM}}} \end{pmatrix} =&\frac{{\textrm{i}}{c_{\textrm{h}}}{Z_{\textrm{h}}}{k_{\textrm{h}}^3}}{6\pi } \begin{pmatrix} {\textbf{C}}\left( \varvec{\alpha }_{\textrm{ee}}\right) {\textbf{C}}^{-1}&{}{\textbf{C}}\left({ -\textrm{i}}{\varvec{\alpha }_{\textrm{em}}}/{Z_{\textrm{h}}}^{}\right) {\textbf{C}}^{-1}\\ {\textbf{C}}\left( {\textrm{i}}{\varvec{\alpha }_{\textrm{me}}}/{c_{\textrm{h}}}^{}\right) {\textbf{C}}^{-1}&{\textbf{C}}\left({ \varvec{\alpha }_{\textrm{mm}}}/({c_{\textrm{h}}}^{}{Z_{\textrm{h}}})\right) {\textbf{C}}^{-1} \end{pmatrix}\,. \end{aligned} \end{aligned}$$

In the previous equation, the $\varvec{\alpha }_{xx}$ are 3$\times$3 complex dynamic polarisability tensors of molecules obtained by quantum chemistry calculations, the ${\textbf{C}}$ is a unitary matrix that translates a *T*–matrix from the Cartesian basis to the spherical basis. The ${{c_{\textrm{h}}}}=1/\sqrt{{{\varepsilon _{\textrm{h}}}}{{\mu _{\textrm{h}}}}}$, in general, is the speed of light in the surrounding medium of the object, and ${Z_{\textrm{h}}}=\sqrt{{\mu _{\textrm{h}}}/{\varepsilon _{\textrm{h}}}}$ is the wave impedance^16^. However, one needs to keep in mind that our quantum chemistry calculations already consider the surrounding medium (water) through the Conductor–like Screening Model. Thus, here we used ${c_{\textrm{h}}}$ = $c_{0}$ to construct the *T*–matrices to accommodate this aspect. These *T*–matrices are the prime quantities used in full–wave electrodynamic scattering simulations to gain insights into the light–matter interactions at relativistic velocities. All experimentally observable properties, such as absorption cross–sections or the circular dichroism, can be obtained from such a *T*–matrix. The detailed theoretical description of the *T*–matrix construction approach from dynamic polarisabilities was presented previously^16–18^.

Finally, we note that the *T*–matrix expresses the relation between the incident and the scattered fields. In the scenario we consider, however, we find considering the outgoing field to be more favourable, since the CD is usually defined with respect to this^39^. Also, as the TCD is measured in the forward direction, it contains information from the scattered and the incident field, and what is measured corresponds exactly to the outgoing field in the forward direction. The aforementioned relation between the incident and the outgoing fields is obtained by using the *T*–matrix to obtain the scattered field, along with adding the contribution of the incoming field, which can be seen in and is explained immediately after Eq. (17).

### Description of the scattering scenario

**Figure 1:**
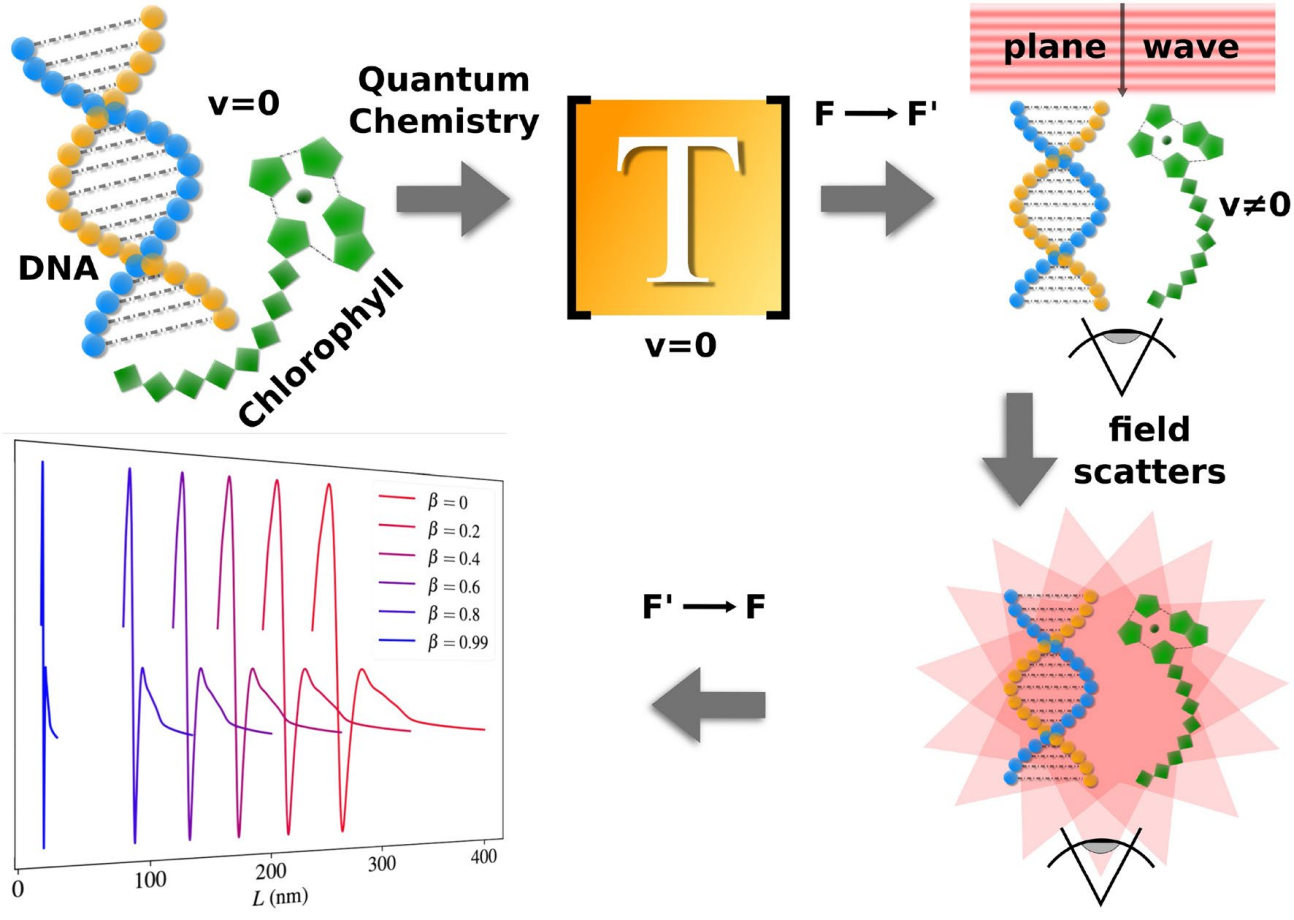
All steps required to obtain the CD in the reference frame *F*. Firstly, the molecules are constructed, and their *T*–matrices are computed using TURBOMOLE code. When describing the actual scattering process, the incident plane wave propagating in the $-z$–direction is first boosted to $F'$, where the outgoing field is obtained by adding the contribution of the incoming field to the scattered field, which is present in and justified directly after Eq. (17). Finally, the outgoing field is inverse boosted back to *F*, where the CD is observed.

Before discussing any mathematical methods, it is important to draw a qualitative picture of our approach to describe the scattering in a relativistic setting. All procedures carried out are illustrated in Fig. 1.

We begin by defining the two frames of reference, that is, the lab frame *F* and the molecules’ frame $F'$. Frame *F* contains the source of the illumination as well as the observer. This setup is analogous to the one presented in Ref. ^12^ where an interstellar chiral molecule, specifically propylene oxide, was discovered for the first time. There, the light source was the giant molecular gas cloud Sagittarius B2 (Sgr B2).

In *F*, the observer witnesses the molecule moving with speed *v* along the negative *z*–axis away from the light source, which is likewise located along this axis. We assume the incoming light to be a plane wave of pure helicity that propagates in the $-z$–direction (see Fig. 1). We utilise the helicity basis because the helicity of electromagnetic fields is invariant under Lorentz boosts^19,40^.

The quantity we wish to investigate is the TCD rotationally–averaged with respect to the incident field. In the stationary case, we can obtain the rotationally–averaged TCD in two ways. The first way would be to illuminate the molecule in a fixed position from multiple angles with plane waves of uniform spacing with respect to each other^41^. Another way would be to illuminate with a fixed plane wave and rotate the *T*–matrix of the molecule using equation (5.29) from Mishchenko et al.^42^, again making sure the incident plane wave illuminates the molecule at uniformly–separated orientations. Both approaches provide identical results, and one of them can be chosen for numerical convenience. In this article, we consider the latter model where we rotate the molecule itself.

Below, we show in Eq. (6) the boosting of an incident field. We firstly boost the incident field into the frame of the molecule, followed by obtaining the scattered and outgoing fields. The outgoing and incident fields are linked, as mentioned, by the *T*–matrix along with a contribution from the incoming field, which we outline in a subsequent section where we determine the TCD in the lab frame. Next, one needs to sum the TCD values for each angle of orientation of the incoming field and divide this sum by the number of orientations. To obtain a reliable result, we consider 50, equally spaced orientation angles in the molecules’ frame. Although the two approaches to find the TCD mentioned above are identical, we use the latter where the molecule itself is rotated instead of the incoming field, since it makes more physical sense in our scenario. It will almost certainly be the case that molecules with random orientations are found in clusters, which, given the long distance between the light source and the cluster, can give rise to a rotationally averaged response. To make sure the orientations are uniformly spaced, we utilise a sampling optimisation algorithm based on gradient descent^43^.

Regarding the boosting procedure, we employ the frame–hopping method, which is outlined as follows:^14,15^

1. The incident field is Lorentz–boosted from *F* to $F'$. The boosted incoming field is obtained from the boosted incident field.
2. The scattered field is obtained in $F'$ using the *T*–matrix.
3. The scattered field is inverse–boosted back to *F*, after which the outgoing field is obtained. Here, the TCD is observed.

Note that we consider active Lorentz boosts like in Refs. ^19^ and ^20^ instead of passive ones. The fields are boosted with the former, whereas the reference frames are boosted with the latter. We opt for the active description, since this explicitly shows us how the fields physically change, whereas the passive transformation only involves relabelling the relevant quantities to fit a new coordinate system.

### Boosting the incident field into the molecules’ frame

Armed with a visual understanding of the problem, we can now build a mathematical description. We note that all quantities pertaining to frames $F'$ and *F* are denoted with and without a prime, respectively. Moreover, the incident field and all quantities linked to it will be denoted with the subscript ‘i’. Beginning with expanding the incident field ${\textbf{E}}_{\textrm{i}}({\textbf{r}}, t)$ with corresponding wave vector ${{\textbf{k}}_{\textrm{i}}}$ in the plane wave basis with expansion coefficients ${f_{\lambda _{\textrm{i}}}}({\textbf{k}})$, we have in *F* that

2 $$\begin{aligned} {\textbf{E}}_\text{i}({\textbf{r}}, t)&= \sum _{\lambda _{\text{i}}=\pm 1}E_{\text{i},\, \lambda _{\text{i}}}\hat{{\textbf{e}}}_{\lambda _{\text{i}}}(\hat{{\textbf{k}}})\text{e}^{-\text{i}(ckt - {\textbf{k}}\cdot {\textbf{r}})}\\&= \sum _{\lambda _{\text{i}}=\pm 1}\int _{{\mathbb {R}}^{3}}\frac{\text{d}^{3}{\textbf{k}}}{k}\,\hat{{\textbf{e}}}_{\lambda _{\text{i}}}(\hat{{\textbf{k}}})f_{\lambda _{\text{i}}}({\textbf{k}})\,k\text{e}^{-\text{i}(ckt - {\textbf{k}}\cdot {\textbf{r}})}\\&\equiv \sum _{\lambda _{\text{i}}=\pm 1}\int _{{\mathbb {R}}^{3}}\frac{\text{d}^{3}{\textbf{k}}}{k}f_{\lambda _{\text{i}}}({\textbf{k}}){|{\lambda _{\text{i}}\,{\textbf{k}}}\rangle }\\&= {|{{\textbf{E}}_{\text{i}}}\rangle }, \end{aligned}$$

where, for simulation purposes, ${E_{\textrm{i}, \,\lambda _{\textrm{i}}}}=1$ for an illumination of pure helicity ${\lambda _{\textrm{i}}}=1$ or ${\lambda _{\textrm{i}}}=-1$. Also, ${\textbf{k}}=|k|(\hat{{\textbf{x}}}\sin \theta \cos \phi + \hat{{\textbf{y}}}\sin \theta \sin \phi + \hat{{\textbf{z}}}\cos \theta )$, $\theta$ and $\phi$ are the polar and azimuthal angles, respectively, and

3 $$\begin{aligned} {\hat{{\textbf{e}}}_{\lambda _{\textrm{i}}}}(\hat{{\textbf{k}}})&= \frac{{-\lambda _{\textrm{i}}}{\hat{\theta }}({\hat{{\textbf{k}}})-\textrm{i}}{\hat{\phi }}(\hat{{\textbf{k}}})}{\sqrt{2}} \end{aligned}$$

is the helical polarisation vector with spherical polar and azimuthal unit vectors ${\hat{\theta }}$ and ${\hat{\phi }}$, respectively, which correspond to $\hat{{\textbf{k}}}={\textbf{k}}/|{\textbf{k}}|$. Furthermore, we have for a single plane wave that

4 $$\begin{aligned} {f_{\lambda _{\textrm{i}}}}({\textbf{k}})&= \frac{1}{k^{2}}\delta (k-{k_{\textrm{i}}})\delta (\cos \theta -\cos {\theta _{\textrm{i}}})\delta (\phi -{\phi _{\textrm{i}}}), \end{aligned}$$

where ${\theta _{\textrm{i}}}=\pi$ and ${\phi _{\textrm{i}}}=0$ and $\delta$ represents the Dirac delta function. Moreover, *c* is the speed of light in a vacuum, ${\textbf{r}}$ is the position vector, ${k_{\textrm{i}}}=|{\textbf{k}}_{\textrm{i}}|$, *t* is time and, using Dirac notation, we have the ket

5 $$\begin{aligned} {|{{\lambda _{\textrm{i}}}\,{\textbf{k}}}\rangle }&\equiv k\hat{{\textbf{e}}}_{{\lambda _{\textrm{i}}}}(\hat{{\textbf{k}}}){\textrm{e}^{-\textrm{i}(ckt - {\textbf{k}}\cdot {\textbf{r}}})}. \end{aligned}$$

Note the unconventional prefactor *k* in (5). This is present to conform with the Lorentz invariant integration measure ${\textrm{d}^{3}}{\textbf{k}}/k$^19,44^ used when computing the electric field.

As mentioned, we consider a plane wave that propagates in the $-z$–direction, which means that ${{\textbf{k}}_{\textrm{i}}}=-{k_{\textrm{i}}}\hat{{\textbf{z}}}$.

We now need to Lorentz–boost the incident field from *F* to $F'$ using the boosting operator $\hat{{\textbf{L}}}_{z}(\beta )$ for a boost along the *z*–axis, where $\beta =v/c$,  $0\le \beta <1$ and $\hat{{\textbf{L}}}_{z}(\beta )$ is defined in Appendix B in Ref. ^19^.

In doing this, one obtains^19^

6 $$\begin{aligned} {|{{{\textbf{E}}_{\textrm{i}}'}}\rangle }&= \hat{{\textbf{L}}}_{z}(\beta ){|{{{\textbf{E}}_{\textrm{i}}}}\rangle }\nonumber \\&= \sum _{{\lambda _{\textrm{i}}'=\pm 1}}\delta _{\lambda _{\textrm{i}}\lambda _{\textrm{i}}'}\int _{{\mathbb {R}}^{3}}\frac{\textrm{d}^{3}{\textbf{k}}}{k}f_{\lambda _{\textrm{i}}}({\textbf{k}}){|{\lambda _{\textrm{i}}'\,\,\hat{{\textbf{L}}}_{z}(\beta ){\textbf{k}}}\rangle }\nonumber \\&= \frac{1}{{k_{\textrm{i}}}}\sum _{\lambda _{\textrm{i}}'=\pm 1}{\delta _{\lambda _{\textrm{i}}\lambda _{\textrm{i}}'}}{|{\lambda _{\textrm{i}}'\,\,\hat{{\textbf{L}}}_{z}(\beta ){\textbf{k}}_{\textrm{i}}}\rangle }, \end{aligned}$$

where $\hat{{\textbf{L}}}_{z}(\beta ){{\textbf{k}}_{\textrm{i}}}={{\textbf{k}}_{\textrm{i}}}(\cosh \xi -\sinh \xi )$ and $\xi = {\textrm{artanh}}(\beta )$. Recall that ${\lambda _{\textrm{i}}'}={\lambda _{\textrm{i}}}$ due to the invariance of helicity under Lorentz boosts^40^, hence the Kronecker delta in Eq. (6). Moreover, as previously mentioned, we consider active boosts and not passive ones. These can be mapped onto each other via the transformation $\beta \rightarrow -\beta$.

### Solving the scattering problem in the molecules’ frame

We are now able to obtain the outgoing field in $F'$. To do this, we begin by expanding the incident field ${{|{\mathbf {E_{\textrm{i}}'}}\rangle }}$ in the spherical wave basis as follows:^19^

7 $$\begin{aligned} {|{\mathbf {E_{\textrm{i}}'}}\rangle }&= \int ^{\infty }_{0}{\textrm{d}}k'\,k'\sum _{\lambda '_{\textrm{i}}=\pm 1}\sum _{\ell '=1}^{\infty }\sum _{m'=-\ell '}^{\ell } A '_{\ell ' m'{\lambda '_{\textrm{i}}}}(k')\delta _{\lambda '_{\textrm{i}}{\lambda _{\textrm{i}}}}{|{k'\,\ell '\,m'\,{\lambda _{\textrm{i}}'}}\rangle }\,, \end{aligned}$$

where, for a plane wave propagating in the $-z$–direction, $A '_{\ell ' m'{\lambda '_{\textrm{i}}}}(k')$ are expansion coefficients given by

8 $$\begin{aligned} A '_{\ell ' m'{\lambda '_{\textrm{i}}}}(k')&= \sqrt{\frac{2\ell '+1}{4\pi }}\int ^{2\pi }_{0}{\textrm{d}}\phi '\int ^{1}_{-1}{\textrm{d}}(\cos \theta ') D^{\ell '}_{m'{\lambda '_{\textrm{i}}}}(\phi ', \theta ', 0)f_{\lambda '_{\textrm{i}}}({\textbf{k}}')\nonumber \\&= \sqrt{\frac{2\ell '+1}{4\pi }}D^{\ell '}_{m'{\lambda '_{\textrm{i}}}}(0, \pi , 0) \frac{1}{k'^{2}}\delta (k'-k_{\textrm{i}}'), \end{aligned}$$

with multipolar index $\ell '$ ($\ell '=1$ corresponds to dipoles, $\ell '=2$ corresponds to quadrupoles, etc.), and angular momentum along the *z*–axis $m'=-\ell ',-(\ell '-1),\hspace{1mm}...\hspace{1mm},\ell '$. Also, $D^{\ell }_{m\lambda }(\phi , \theta , \psi )$ is the Wigner–D matrix element given by^42^

9 $$\begin{aligned} D^{\ell }_{m\lambda }(\phi , \theta , \psi )&= {\textrm{e}^{-im\phi} }d^{\ell }_{m\lambda }(\theta ){\textrm{e}^{-\textrm{i}\lambda \psi }}, \end{aligned}$$

where $d^{\ell }_{m\lambda }(\theta )$ are the Wigner d–functions defined in^45^ Sec. 7.3. and $(\theta , \phi , \psi )$ are Euler angles. Moreover, the spherical wave basis ket ${|{k'\,\ell '\,m'\,{\lambda _{\textrm{i}}'}}\rangle }$ is given by^19^

10 $$\begin{aligned} {|{k'\,\ell '\,m'\,\lambda _{\textrm{i}}'}\rangle }&= \sqrt{\frac{2\ell '+1}{4\pi }}\int ^{2\pi }_{0}\textrm{d}\phi '\int ^{1}_{-1}\textrm{d}(\cos \theta ') D^{\ell '}_{m'\lambda '_{\textrm{i}}}(\phi ', \theta ', 0)^{*}{|{\lambda _{\textrm{i}}'\, -k'\hat{{\textbf{z}}}}\rangle }. \end{aligned}$$

Since our molecules are of nanometre scale, one only needs to consider the dipole component of ${|{\mathbf {E_{\textrm{i}}'}}\rangle }$, that is, when $\ell =1$. As mentioned, the contribution of the incoming field to the outgoing field is included after obtaining the scattered field. Note that, to transform from the plane wave to the spherical wave basis, we utilised the Python package *treams*^46^, specifically the function treams.pw.to_sw.

The next step is to determine the outgoing field in $F'$. This is done by first obtaining the scattered field using ${\textbf{T}}$, which relates the scattered field to the incident field^42^.

With this in mind, the scattered field is given by

11 $$\begin{aligned} {|{\mathbf {E_{\textrm{s}}'}}\rangle }&= \int ^{\infty }_{0}{\textrm{d}}k'\,k'\sum _{\lambda '_{\textrm{s}}=\pm 1}\sum _{\ell '=1}^{\infty }\sum _{m'=-\ell '}^{\ell } B '_{\ell ' m'{\lambda '_{\textrm{s}}}}(k'){|{k'\,\ell '\,m'\,{\lambda _{\textrm{s}}'}}\rangle }\,, \end{aligned}$$

where $B '_{\ell ' m'{\lambda '_{\textrm{s}}}}(k')$ are the scattered field expansion coefficients and the subscript ‘s’ denotes quantities relating to the scattered field. These coefficients are related to the incident expansion field coefficients in matrix form in the following way:

12 $$\begin{aligned} {\textbf{B}}^{'+}={{\textbf{T}}^{\textrm{H}}}{\textbf{A}}^{'+}\,\,\,\text {and}\,\,\, {\textbf{B}}^{'-}={{\textbf{T}}^{\textrm{H}}}{\textbf{A}}^{'-}\, \end{aligned}$$

where

13 $$\begin{aligned} {\textbf{A}}^{'+} = \begin{pmatrix} {\textbf{A}}^{'+}_{3}\\ {\textbf{0}}_{3} \end{pmatrix} \,\,\,\text {and}\,\,\, {\textbf{A}}^{'-} = \begin{pmatrix} {\textbf{0}}_{3}\\ {\textbf{A}}^{'-}_{3}, \end{pmatrix} \end{aligned}$$

are 6 by 1 vectors containing the components of ${\textbf{A}}'$ for an incident wave of pure positive and pure negative helicity, respectively. The ‘H’ superscript denotes the *T*–matrix in the helicity basis, which is converted from the parity basis using a dedicated function in *treams*. The vectors ${\textbf{A}}^{'+}_{3}$ and ${\textbf{A}}^{'-}_{3}$ are 3 by 1 vectors containing the positive and negative helicity components of ${\textbf{A}}'$, respectively, and ${\textbf{0}}_{3}$ is the 3 by 1 zero vector. Note that we require that ${\textbf{A}}^{'+}$ and ${\textbf{A}}^{'-}$ have their aforementioned dimension since we consider a 6 by 6 *T*–matrix, that is, of dipolar order.

As previously mentioned, we wish to determine the rotationally–averaged TCD by illuminating a molecule with 50 different, equally spaced angles of orientation $\theta '\in [0, \pi )$ and $\Phi '\in [0, 2\pi )$. This is equivalent to applying a rotated *T*–matrix ${{\textbf{T}}^{\textrm{H}}_{\textrm{R}}}(\theta ', \Phi ')$ to ${\textbf{A}}^{'+}$ and ${\textbf{A}}^{'-}$, where

14 $$\begin{aligned} {{\textbf{T}}^{\textrm{H}}_{\textrm{R}}}(\theta ', \Phi ')&= {\textbf{R}}(\theta ', \Phi '){{\textbf{T}}^{\textrm{H}}}{\textbf{R}}^{-1}(\theta ', \Phi ') \end{aligned}$$

and ${\textrm{R}}(\theta ', \Phi ')$ is a rotation matrix in $F'$ applied using the *treams* function treams.rotate. The angles *θ* and Φ correspond to Euler angles representing a rotation about the y-axis and the z-axis, respectively. Note that we omit an initial rotation about the z-axis, as is commonly done with Euler angles, since our incident field is a circularly-polarised plane wave in the x-y plane. In this case, an initial rotation about the z-axis would only produce a phase term, which would cancel out when later taking its modulus squared upon determining the relevant intensities.

As a result, we have for each angle of orientation

15 $$\begin{aligned} {\textbf{B}}'^{\pm }(\theta ', \Phi ')&={ {\textbf{T}}^{\textrm{H}}_{\textrm{R}}}(\theta ', \Phi '){\textbf{A}}'^{\pm }. \end{aligned}$$

Since the rotational averaging is carried out in $F'$, we emphasise the requirement that the angles of orientation be equally spaced in $F'$ and not in *F*.

**Figure 2:**
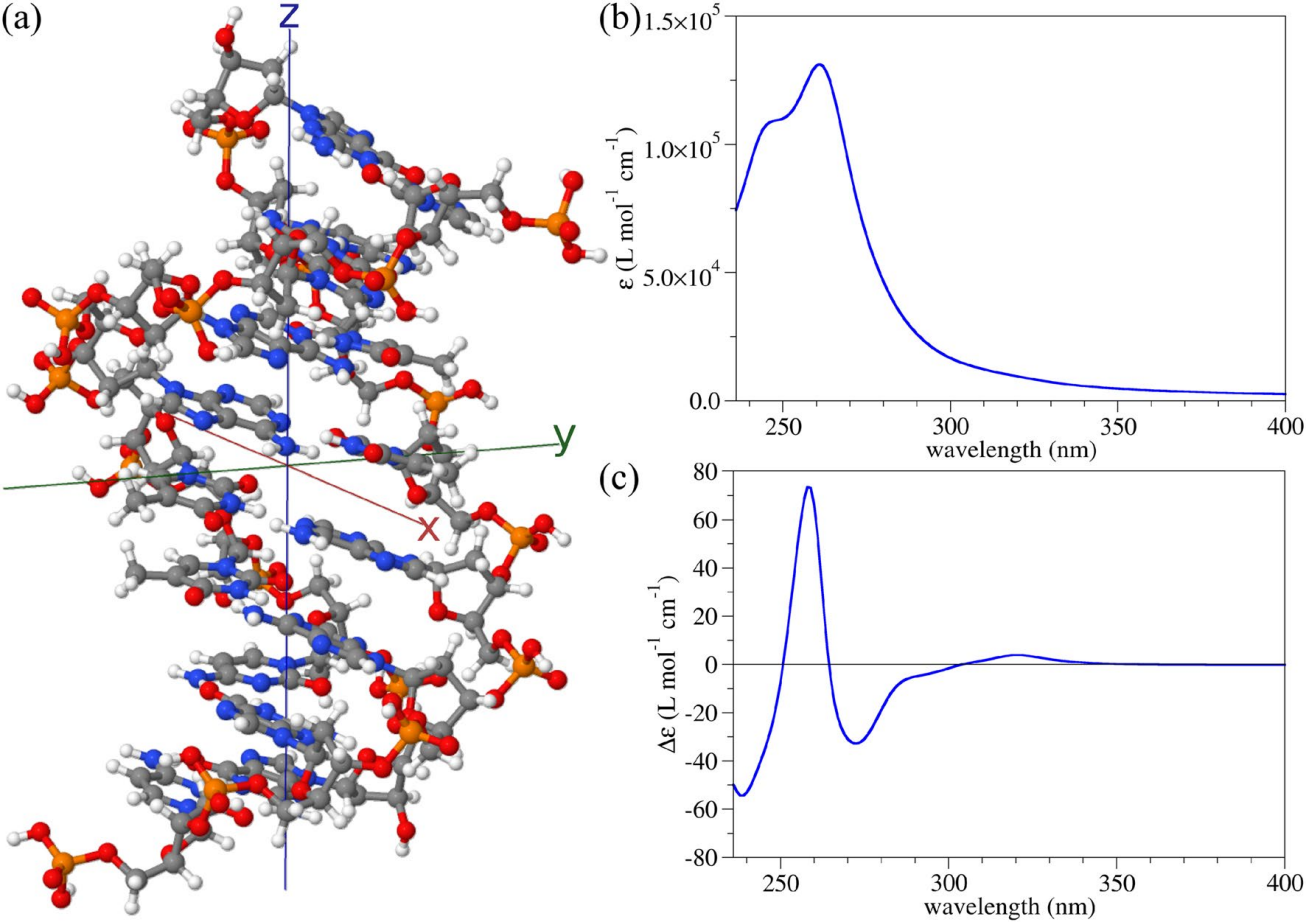
(**a**) A finite–size molecular model of B–DNA containing eight DNA base pairs. White, grey, blue, red, and orange balls represent hydrogen, carbon, nitrogen, oxygen, and phosphorus atoms, respectively. (**b**) UV molar absorption spectrum reconstructed from dynamic polarisabilities. A Lorentzian broadening of 0.15 eV at HWHM was used. (**c**) UV ACD molar spectrum reconstructed from dynamic polarisabilities. The same broadening as in (**b**) was used.

### Inverse boosting the circular dichroism to the lab frame

Now that we have the expansion coefficients ${\textbf{B}}'^{\pm }(\theta ', \Phi ')$ of the scattered field in $F'$, we can now begin with determining the TCD in *F*. Specifically, we define the rotationally–averaged TCD in *F* viewed along the $-z$-direction, where ${\theta '_{\textrm{s}}}={\theta _{\textrm{s}}}=\pi$. To do this, we firstly convert the coefficients ${\textbf{B}}'^{\pm }(\theta ', \Phi ')$ to ones in the plane wave basis denoted ${E'^{+}_{\textrm{s},\, \lambda _{\textrm{s}}}=\pm 1}$ and ${E'^{-}_{\textrm{s},\, \lambda _{\textrm{s}}}=\pm 1}$, which are analogous to the coefficients ${E_{\textrm{i}, \,\lambda _{\textrm{i}}}}$ found in Eq. (2). Moreover, the $+$ and − superscripts refer to the coefficients which relate to an incident field of pure positive or negative helicity, respectively, and

16 $$\begin{aligned} {E_{\textrm{s},\, {\lambda _{\textrm{s}}}=\pm 1}'^{\pm }}&= -\sqrt{2}{E'^{\pm }_{\textrm{s},\, \lambda _{\textrm{s}}=\pm 1, \theta '_{\textrm{s}=\pi }}}, \end{aligned}$$

where ${E'^{\pm }_{\textrm{s},\, \lambda _{\textrm{s}}=\pm 1, \theta '_{\textrm{s}=\pi }}}$ are the polar components of the scattered field defined in equations (4) and (5) in the supplementary information which are obtained by taking the far-field limit of equations (11a) and (11b) in Ref. ^46^. Despite the appearance of the ${\textrm{e}^{\textrm{i}k'{\textrm{s}}r'}}/{k'_{\textrm{s}}}r'$ term in equations (4) and (5) in the supplementary information, we assume that the molecules are far enough from the observer ($r'>>0$) such that $1/{k'_{\textrm{s}}}r'$ varies slowly, meaning our scattering coefficients can be treated as plane wave coefficients. Moreover, one simply sees as a result, a scaling in the magnitude of the TCD as $r'$ increases. Since we are only interested in the sign of the TCD, we omit the $1/{k'_{\textrm{s}}}r'$ contribution and consider an arbitrary scaling in magnitude.

Finally, using the approach from Ref. ^20^, we boost the coefficients ${E_{\textrm{s},\, \lambda _{\textrm{s}}=\pm 1}'^{\pm }}$ from $F'$ to *F* to obtain ${E_{\textrm{s},\, \lambda _{\textrm{s}}=\pm 1}^{\pm }}$. Here, we inverse boost the plane wave coefficients ${E_{\textrm{s},\, \lambda _{\textrm{s}}=\pm 1}'^{\pm }}$ and not the spherical wave coefficients ${\textbf{B}}'^{\pm }(\theta ', \Phi ')$. If we were to remain in the spherical wave basis, we would have to account for additional multipolar orders acquired due to the mixing of fields under the Lorentz boost^47^. In this case, one would be required to truncate an infinite sum of multipoles in *F* when determining the TCD, thus significantly increasing computation time and decreasing the precision of the final result. Moreover, the order at which one would need to truncate the sum of the multipoles would increase with speed, since the mixing of the fields would become more intense. By converting to the plane wave basis before boosting, we omit the need to consider infinite sums and, as a result, retain all information about the scattered field.

To determine the TCD, we finally need to calculate the transmission $T^{+}$ due to an incoming field with pure positive helicity and one with pure negative helicity, $T^{-}$, where,

17 $$\begin{aligned} T^{+} = |{E^{+}_{\textrm{s},\, \lambda _{\textrm{s}}=1}} + {E_{\textrm{in},\, \lambda _{\textrm{i}=1}}}|^{2}+|E^{+}_{\textrm{s},\, \lambda _{\textrm{s}}=-1}|^{2}\,\,\,\text {and}\,\,\,T^{-} = |{E^{-}_{\textrm{s},\, \lambda _{\textrm{s}}=1}}|^{2}+|{E^{-}_{\textrm{s},\, \lambda _{\textrm{s}}=-1}}+{E_{\textrm{in},\, \lambda _{\textrm{i}=-1}}}|^{2}, \end{aligned}$$

where the incoming fields ${E_{\textrm{in},\, \lambda _{\textrm{i}=1}}}$ and ${E_{\textrm{in},\, \lambda _{\textrm{i}=-1}}}$ are present for ${E^{+}_{\textrm{s},\, \lambda _{\textrm{s}}=1}}$ and ${E^{-}_{\textrm{s},\, \lambda _{\textrm{s}}=-1}}$ including also the higher-order components of the incoming field which do not interact with the molecule, but still contribute to the outgoing field, and hence the TCD. Using Eq. (17), we calculate the TCD as follows:

18 $$\begin{aligned} {\textrm{TCD}}&={ \frac{T^{-} - T^{+}}{T^{-} + T^{+}}}\,. \end{aligned}$$

The previous equation defining the TCD is the most important equation in the paper and the quantity whose variation we will investigate under Lorentz boosts.

## Results and discussion

### Molar absorption and absorption circular dichroism (ACD) spectra

**Figure 3:**
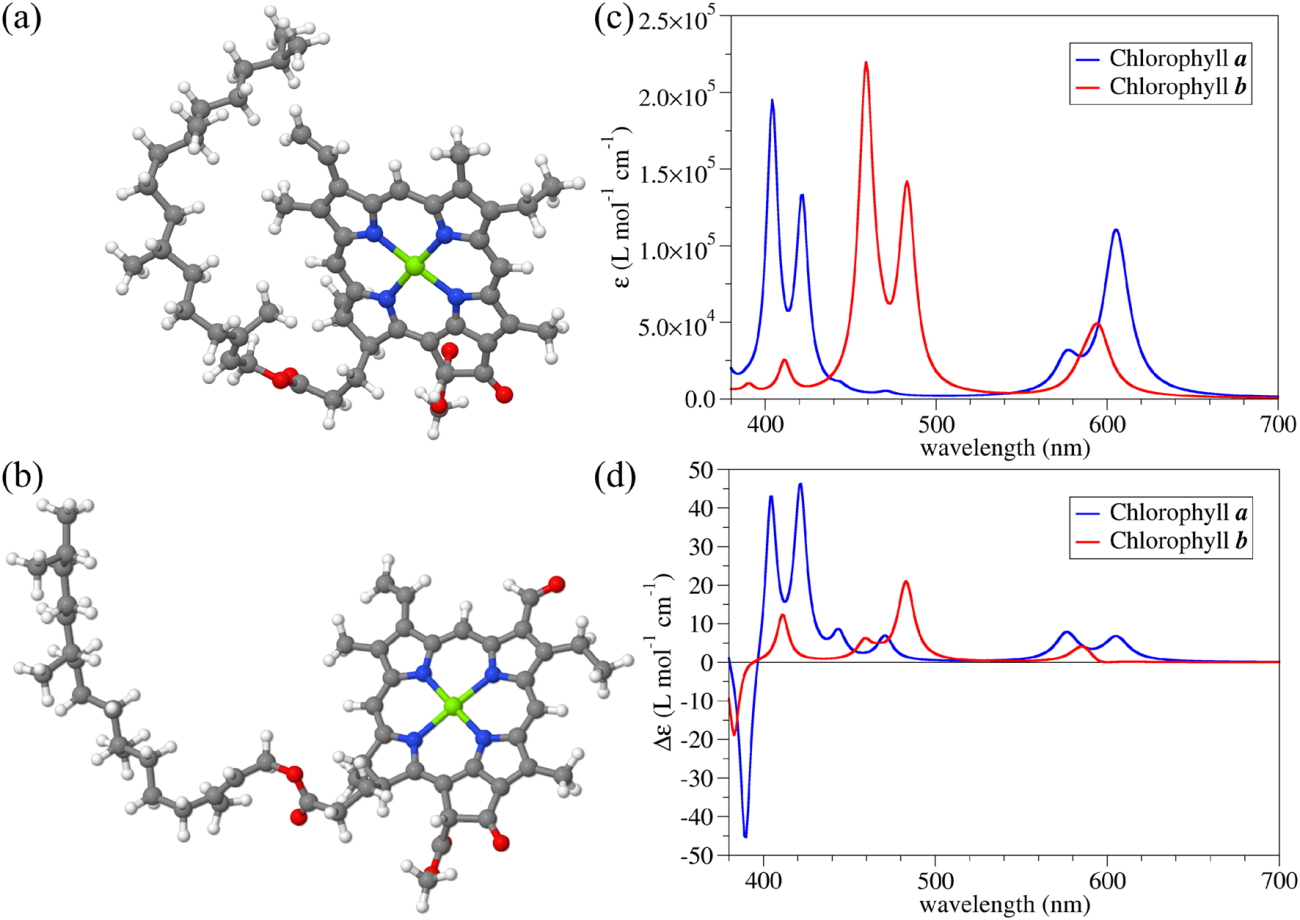
Optimised geometries of (**a**) chlorophyll *a* and (**b**) chlorophyll *b* in a COSMO model for water. White, grey, blue, red, and green balls represent hydrogen, carbon, nitrogen, oxygen, and magnesium atoms, respectively. (**c**) UV molar absorption spectra of both chlorophyll molecules reconstructed from dynamic polarisabilities. A Lorentzian broadening of 0.03 eV at HWHM was used. (**d**) UV ACD molar spectra of chlorophyll *a* and *b* reconstructed from dynamic polarisabilities. The same broadening as in (**c**) was used.

Before discussing relativistic effects, we first present the results of quantum chemistry simulations for static B–DNA, chlorophyll *a*, and chlorophyll *b* molecules. Specifically, we show the rotationally-averaged absorption and absorption circular dichroism (ACD) for each molecule. We provide these results as a reference point, since the CD is usually defined with respect to absorption. This will also become important when we later consider the transmission circular dichroism (TCD).

The geometry of the finite–size B–DNA molecular model used here is presented in Fig. 2a and has a reduced sequence of eight base pairs (5’–CGAATTCG–3’) extracted from the crystallographic file.^48^ The linear absorption spectrum of this DNA model in an aqueous surrounding is presented in Fig. 2b, while its ACD spectrum is depicted in Fig. 2c. Comparison of these simulations with experimental results from the literature shows a good agreement,^49–52^ giving us confidence that the size of the selected B–DNA model is sufficiently large to capture all relevant optical transitions in the complete DNA molecule.

Further, we look into the optical properties of single chlorophyll *a* and *b* biomolecules in an implicit water surrounding. Initial geometries of the chlorophyll biomolecules were obtained from the CompTox Chemicals Dashboard database^53,54^. Optimised geometries of both molecules are presented in Fig. 3a and b. The molar absorption spectra visualised in Fig. 3c match experimental observations^55^. It is worth noting here that in our quantum chemistry simulations, only electronic transitions were taken into account while vibronic contributions were neglected due to computational complexity. As a result, the lowest–lying peaks in the absorption are blue–shifted compared to experimental measurements. Nevertheless, these two variants of chlorophyll have drastically different absorption values. The only structural difference is the change from the methyl to the formyl group on the chlorine ring^56^. Chlorophyll *b* has been evolutionary modified in plants to absorb more light in the visible part of the electromagnetic spectrum compared to chlorophyll *a*^57^. On the other hand, chlorophyll *a* has a much more intensive ACD signal, especially in the region around 400 nm. Experimental measurements of the ACD of the chlorophyll molecules are in qualitative agreement with our predictions based on a single solvated molecule^58^. It is furthermore noted that the molecular response function is nearly constant with increasing speed. As outlined in the supplementary information using proper relativistic transformations of the electronic Hamiltonian^59,60^, effects of the latter on the results in this or the following section are therefore negligible.

Having the dynamic polarisability tensors of all three biomolecules at hand, the relevant *T*–matrices are used to study relativistic effects on the chiral optical properties in the following results.

### The Lorentz–boosted transmission circular dichroism (TCD)

**Figure 4:**
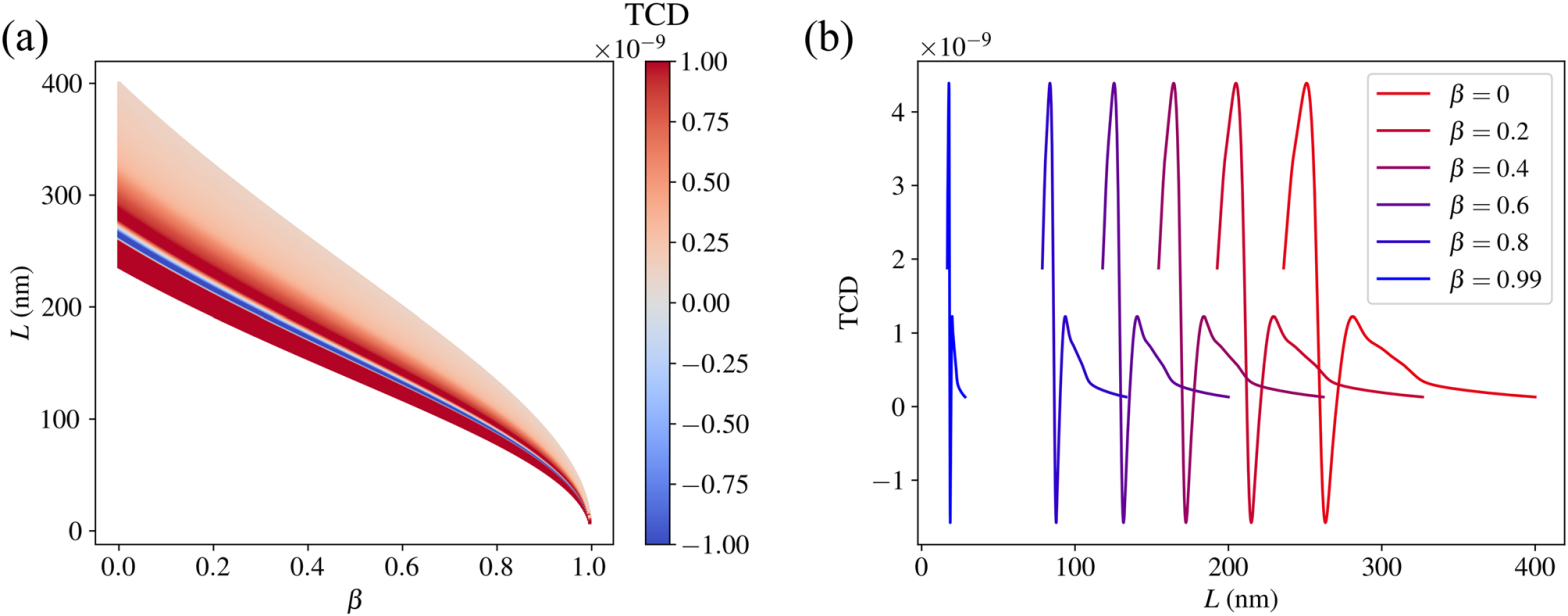
(**a**) The TCD (with arbitrary scaling) of the B–DNA molecule as a function of the speed parameter $\beta$ and the incident wavelengths corresponding to the relevant *T*–matrices. (**b**) The TCD for selected speeds to illustrate the spectral shift.

**Figure 5:**
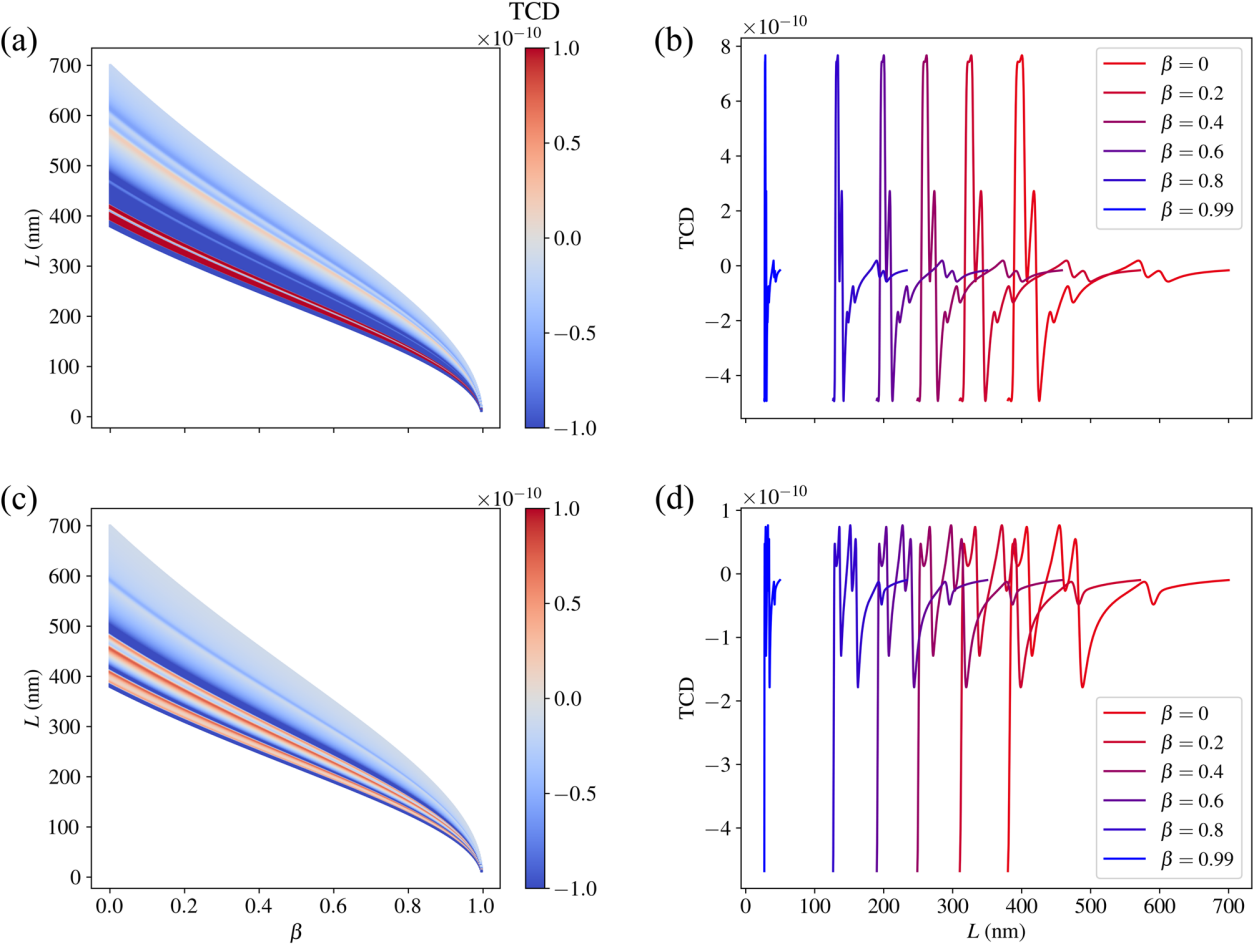
(**a**) The TCD (with arbitrary scaling) of the chlorophyll *a* molecule as a function of the speed parameter $\beta$ and the incident wavelengths corresponding to the relevant *T*–matrices. (**b**) The TCD for selected speeds to illustrate the spectral shift. (**c**) and (**d**) Show the analogous plots for chlorophyll.*b*.

We display our findings as the TCD rotationally–averaged with respect to the incoming field as a function of the wavelength $L=2\pi /k$ in *F* and $\beta =v/c$, where we in each case consider 500 equally–spaced values for $0\le \beta <1$ (cf. Figs. 4 and 5). Note that we use *L* for the wavelength to avoid confusion with the symbol $\lambda$ for helicity. Unlike in the previous section, we now consider the TCD and not the ACD, since the TCD can be evaluated in a specific direction (which, as mentioned, we consider to be the $-z$–direction). A directive approach is much more conducive to accurate results in the relativistic regime, since one would otherwise require detectors in all directions, something that is virtually impossible in outer space. One notices in each plot the varying width of the wavelength spectrum for an increasing speed. This is due to the Doppler shift induced by the Lorentz boost. Conceptually, this is because the incident light, going from the frame *F* to the molecules’ frame $F'$, is shifted to longer wavelengths in $F'$, and the features of the TCD spectrum are imprinted onto the light’s spectrum in $F'$. The observer then detects the light in the original frame *F*, pushing the imprinted features to shorter wavelengths (as the molecule moves towards the observer). Likewise, the Doppler shift causes all the features in the spectra to become narrower (as a function of wavelength) as speed increases. We also note that the plotted wavelength range depends on the speed. This is because the optical properties (the *T*–matrices) were determined only for a limited spectral range, so we can only consider the wavelengths in *F* that, when boosted, are within this limited range in the molecules’ frame $F'$.

In Fig. 4a, we see a colour plot depicting the variation of the TCD for the B–DNA molecule as a function of *L* and $\beta$. As $\beta$ increases, the TCD spectrum shifts to shorter wavelengths to account for the Doppler shift. As $\beta \rightarrow 1$, that is, as the molecule approaches the speed of light *c*, the spectrum for *L* tends to a single point, namely $L=0$. This is because the length contraction of the incoming field in *F* becomes infinitely great. The shift of the spectra is more clearly seen in Fig. 4b, where we see the TCD selected for speeds $\beta \in \{0, 0.2, 0.4, 0.6, 0.8, 0.99\}$. Note that the magnitude of the TCD remains constant for every speed. This is because, under Lorentz boosts, the outgoing field coefficients are only affected by a wavelength scaling given by equation (16) in Ref. ^20^. As a result, the TCD is also only affected by this scaling, which means it both constricts and shifts to lower wavelengths in *F* as the speed increases. Analogous results are also displayed for chlorophyll *a* and *b* in Fig. 5.

It is important to note that the sign of the TCD and the ACD presented in the previous section are not necessarily the same (cf. Fig. 3, 5 and S2 ). This makes sense, since the ACD data considers outgoing fields in all directions, whereas the TCD only considers an outgoing field in the $-z$–direction.

## Conclusions

The main goal of this paper was to demonstrate the effect of relativistic motion on the rotationally–averaged transmission circular dichroism (TCD) of three important biomolecules, namely B–DNA, chlorophyll *a*, and chlorophyll *b*. To do this, we incorporated quantum chemistry calculations by using the TURBOMOLE electronic structure program, from which we could determine the transition (*T*–) matrices of such complicated structures. Moreover, the frame–hopping method was employed to obtain Lorentz–boosted quantities, such as the incoming and outgoing field expansion coefficients. Firstly, an incident field of pure helicity was boosted from the lab (observer’s) frame to the molecules’ frame. Then, the boosted scattered field was obtained using the relevant *T*–matrices. Secondly, for the purpose of rotationally averaging the TCD, we calculated the spherical wave expansion coefficients of the scattered field in the molecules’ frame for 50 different molecular orientations. Next, the expansion coefficients in the spherical wave basis were converted to ones in the plane wave basis, which we then inverse Lorentz boosted from the molecules’ frame to the lab frame using Ref. ^20^. Afterwards, the outgoing field coefficients were determined by adding the contribution of the incoming field to the scattered field in the lab frame. Finally, the transmissions that arise from incoming plane waves of pure positive and negative helicity were calculated, from which the TCD as a function of the molecules’ speed was obtained. Specifically, we noticed a spectral shift to shorter wavelengths with an increase in molecular speed, along with an invariance of the magnitude of the TCD. An invariant magnitude is expected, since the TCD simply scales on the wavelength axis as a function of speed.

Our results make a significant contribution to the topic of detecting chiral molecules in space, which is gaining popularity as demonstrated by the recent work (Ref. ^12^), where the interstellar existence of the chiral molecule propylene oxide was determined for the first time. By extending this area of research by considering biomolecules moving at relativistic speeds, we have added to it a necessary generality. Given the proposed Breakthrough Starshot Initiative^21,22^ where the aim is to accelerate light sails to a speed of 0.2*c*, it is reasonable to assume there exist extraterrestrial structures also moving at this speed or potentially faster, thus opening an area of future practical applications of our work.

## Supplementary Information

Supplementary Information.

## Data Availability

Data is available upon reasonable request from corresponding authors. All results of molecular quantum chemistry calculations can be found deposited to the NOMAD database for materials science under the following 10.17172/NOMAD/2024.02.07-1.
