# Author Correction: Circular dichroism of relativistically–moving chiral molecules

**DOI:** 10.1038/s41598-024-78600-2

**Published:** 2024-12-10

**Authors:** Mitchell R. Whittam, Benedikt Zerulla, Marjan Krstić, Maxim Vavilin, Christof Holzer, Markus Nyman, Lukas Rebholz, Ivan Fernandez-Corbaton, Carsten Rockstuhl

**Affiliations:** 1 Institute of Theoretical Solid State Physics, Karlsruhe Institute of Technology (KIT), Kaiserstr. 12, 76131 Karlsruhe, Germany; 2 Institute of Nanotechnology, Karlsruhe Institute of Technology (KIT), Kaiserstr. 12, 76131 Karlsruhe, Germany

Correction to: *Scientific Reports* 10.1038/s41598-024-66443-w, published online 22 July 2024

The original version of this Article contained errors.

The graphs in Figures 1, 4 and 5 were incorrect, due to an incorrectly implemented function in the code.

The original Figures 1, 4 and 5 and their accompanying legends appear below.

**Fig. 1:**
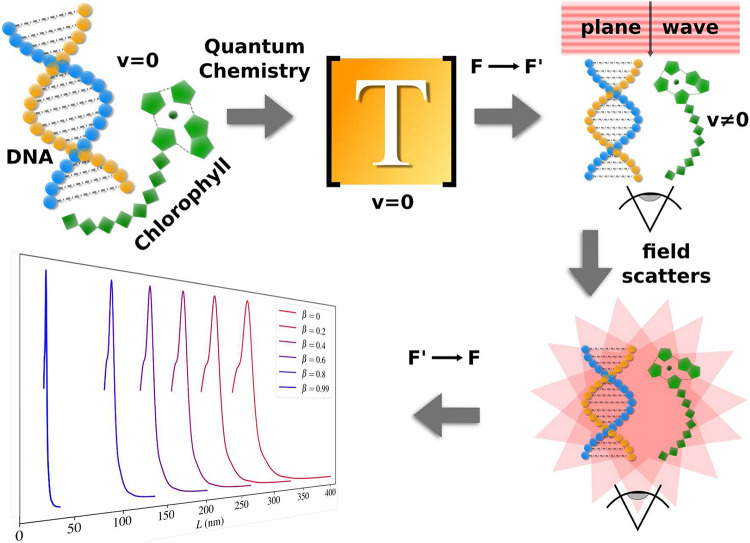
All steps required to obtain the CD in the reference frame F. Firstly, the molecules are constructed, and their T–matrices are computed using TURBOMOLE code. When describing the actual scattering process, the incident plane wave propagating in the − z–direction is first boosted to F′, where the outgoing field is obtained by adding the contribution of the incoming field to the scattered field, which is present in and justified directly after Eq. (17). Finally, the outgoing field is inverse boosted back to F, where the CD is observed.

**Fig. 4:**
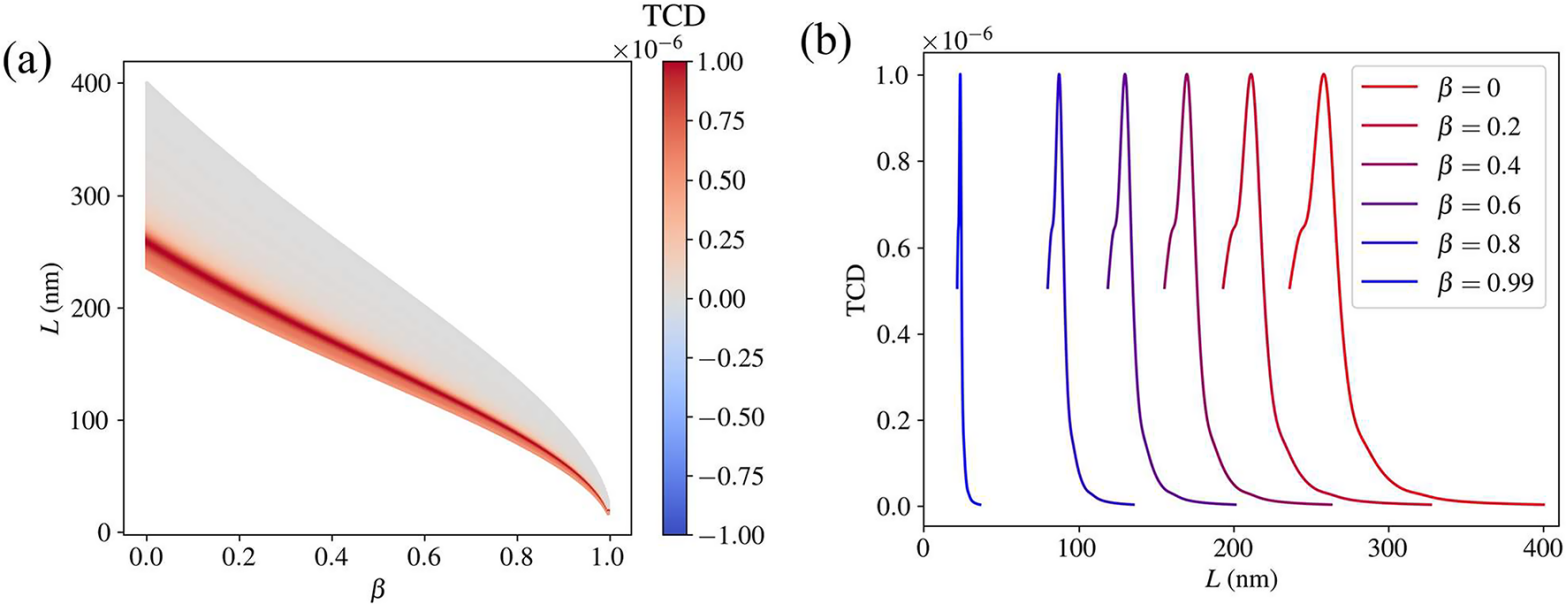
(**a**) The TCD (with arbitrary scaling) of the B–DNA molecule as a function of the speed parameter β and the incident wavelengths corresponding to the relevant *T*–matrices. (**b**) The TCD for selected speeds to illustrate the spectral shift.

**Fig. 5:**
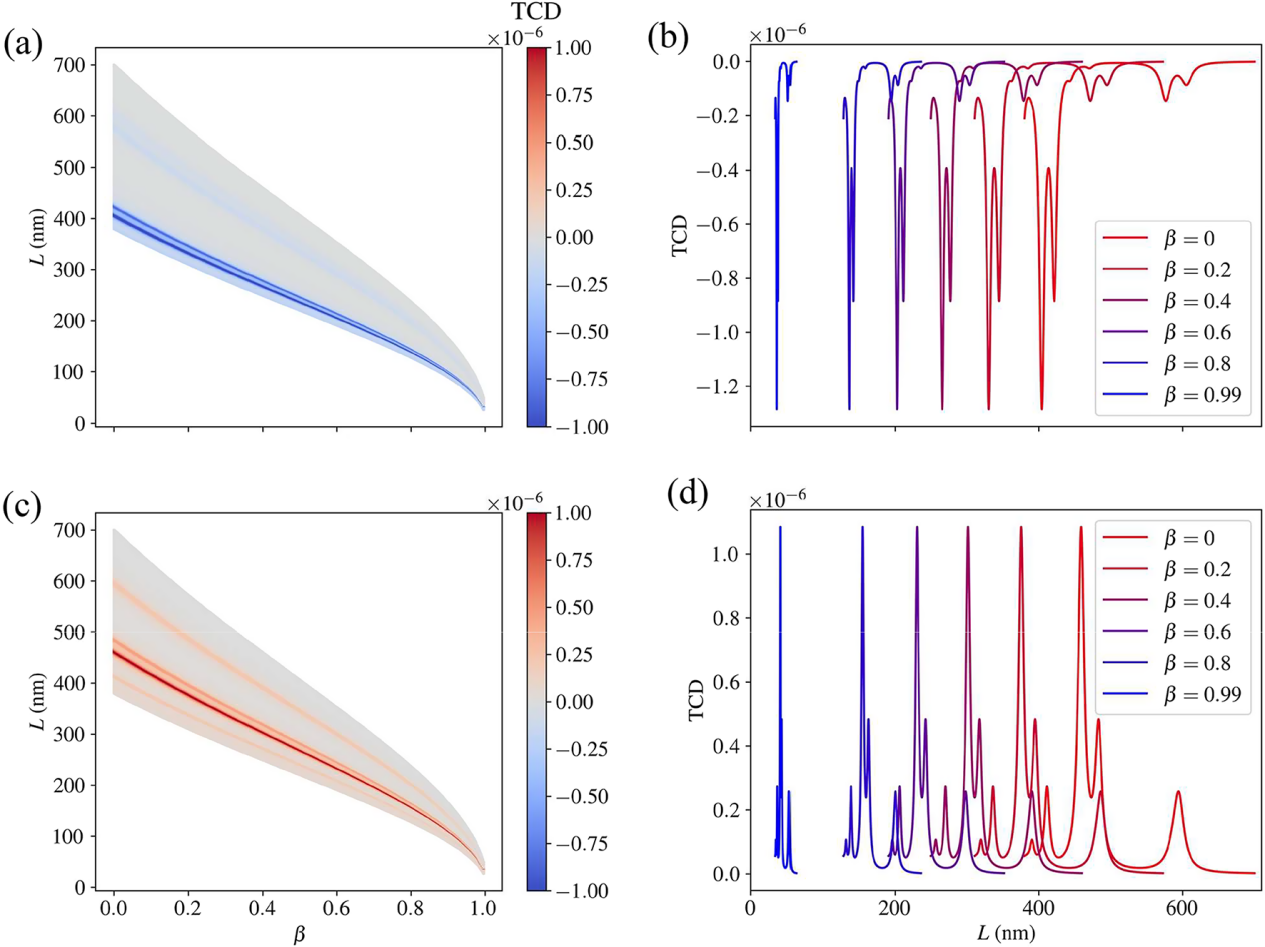
(**a**) The TCD (with arbitrary scaling) of the chlorophyll *a* molecule as a function of the speed parameter $\beta$ and the incident wavelengths corresponding to the relevant *T*–matrices. (**b**) The TCD for selected speeds to illustrate the spectral shift. (**c**) and (**d**) Show the analogous plots for chlorophyll.*b*.

In addition, in the Methods section, under the subheading ‘Solving the scattering problem in the molecules’ frame’,

“As previously mentioned, we wish to determine the rotationally–averaged TCD by illuminating a molecule with 50 different, equally spaced angles of orientation ${\theta }^{\prime}\in [0,\pi )$ and ${\Phi }^{\prime}\in [\text{0,2}\pi )$. This is equivalent to applying a rotated *T*–matrix ${\text{T}}_{\text{R}}^{\text{H}}({\theta }^{\prime},{\Phi }^{\prime})$ to ${\text{A}}^{{\prime}+}$ and ${\text{A}}^{{\prime}-}$, where

14 $$\begin{array}{cc}{\text{T}}_{\text{R}}^{\text{H}}({\theta }^{\prime},{\Phi }^{\prime})& ={\text{R}}({\theta }^{\prime},{\Phi }^{\prime}){\text{T}}^{\text{H}}{{\text{R}}}^{-1}({\theta }^{\prime},{\Phi }^{\prime})\end{array}$$

and ${\text{R}}({\theta }^{\prime},{\Phi }^{\prime})$ is a rotation matrix in ${F}^{\prime}$ applied using the *treams* function ${\texttt{treams.rotate}}$.”

now reads:

“As previously mentioned, we wish to determine the rotationally–averaged TCD by illuminating a molecule with 50 different, equally spaced angles of orientation ${\theta }^{\prime}\in [0,\pi )$ and ${\Phi }^{\prime}\in [\text{0,2}\pi )$. This is equivalent to applying a rotated *T*–matrix ${\text{T}}_{\text{R}}^{\text{H}}({\theta }^{\prime},{\Phi }^{\prime})$ to ${\text{A}}^{{\prime}+}$ and ${\text{A}}^{{\prime}-}$, where

14 $$\begin{array}{cc}{\text{T}}_{\text{R}}^{\text{H}}({\theta }^{\prime},{\Phi }^{\prime})& ={\text{R}}({\theta }^{\prime},{\Phi }^{\prime}){\text{T}}^{\text{H}}{{\text{R}}}^{-1}({\theta }^{\prime},{\Phi }^{\prime})\end{array}$$

and ${\text{R}}({\theta }^{\prime},{\Phi }^{\prime})$ is a rotation matrix in ${F}^{\prime}$ applied using the *treams* function ${\texttt{treams.rotate}}$. The angles $\theta$ and $\Phi$ correspond to Euler angles representing a rotation about the y-axis and the z-axis, respectively. Note that we omit an initial rotation about the z-axis, as is commonly done with Euler angles, since our incident field is a circularly-polarised plane wave in the x–y plane. In this case, an initial rotation about the z-axis would only produce a phase term, which would cancel out when later taking its modulus squared upon determining the relevant intensities.”

The original Article has been corrected.

