# Supplementary material for: Circular dichroism of relativistically–moving chiral molecules
Source: Sci Rep. 2024 Jul 22;14:16812. doi: 10.1038/s41598-024-66443-w (PMC11632085; doi:10.1038/s41598-024-66443-w)
Supplement: Supplementary file 1 — Supplementary Information. [file 41598_2024_66443_MOESM1_ESM.pdf]

Supplementary Information: Circular dichroism of relativistically–moving chiral molecules

Mitchell R. Whittam,[1] Benedikt Zerulla,[2] Marjan Krstić,[1] Maxim Vavilin,[1] Christof Holzer,[1] Markus Nyman,[2] Lukas Rebholz,[1] Ivan Fernandez-Corbaton,[2] and Carsten Rockstuhl[1, 2]

[1)]*Institute of Theoretical Solid State Physics, Karlsruhe Institute of Technology (KIT), Kaiserstr. 12, 76131 Karlsruhe, Germany*

[2)]*Institute of Nanotechnology, Karlsruhe Institute of Technology (KIT), Kaiserstr. 12, 76131 Karlsruhe, Germany*

(Dated: 21 May 2024)

RESPONSE OF A MOLECULAR SYSTEM MOVING AT HIGH VELOCITY

A variant of the relativistic X2C transformation, taking into account the velocity of the system, has been considered to evaluate the molecular response of the system at high velocity. Following Ref. 1, we replace Eq. (14) therein by[1,2]

$$\mathbb{K} = \begin{pmatrix} \mathbf{K} & \mathbf{0} \\ \mathbf{0} & 2c\sqrt{m}\sqrt{\frac{1}{\sqrt{1+\frac{v^2}{c^2}}}}\mathbf{K}p^{-1} \end{pmatrix}. \tag{1}$$

This transformation takes the velocity of the molecule itself into account, yielding the influence thereof on the molecular electron density. Subsequently, the response function is also altered. As shown by Fig S1 for chlorophyll *a*, however, the influence of approaching velocities at a fraction of the speed of light is negligible. There is barely any difference in the molecular response function at different velocities, making the this effect negligible. Assuming the molecular response function to be constant with increasing velocity therefore is an excellent approximation. This assumption can then conveniently be accessed simply from the non-relativistic calculation outlined by $c = \infty$ in Fig S1.

The simulations outlined in Fig. S1 have been carried out using the PBE0 functional[3,4] in combination with the x2c-TZVPPall-2c basis set.[5] A more extended basis set was chosen to ensure a higher flexibility upon the representation of the relativistic molecular transformation.

FAR-FIELD EXPANSION OF SCATTERED VECTOR SPHERICAL WAVES

In this section, we derive the far-field expansion of scattered vector spherical waves $|k'_\mathrm{s}\,\ell'\,m'\,\lambda'_\mathrm{s}\rangle$ used in the main article. Instead of the ket $|k'_\mathrm{s}\,\ell'\,m'\,\lambda'_\mathrm{s}\rangle$, we use the notation $\mathbf{W}^{(3)}_{\ell' m' \lambda'_\mathrm{s}}(\mathbf{r}', k'_\mathrm{s})$ for the scattered vector spherical wave with wave number k'_s and helicity λ'_s. The vector spherical waves of well-defined helicity can be expressed as a sum of vector spherical waves of well-defined parity,

$$\mathbf{W}'^{(3)}_{\ell' m' \lambda'_\mathrm{s}}(\mathbf{r}', k'_\mathrm{s}) = \frac{\mathbf{N}'^{(3)}_{\ell' m'}(\mathbf{r}', k'_\mathrm{s}) + \lambda'_\mathrm{s}\mathbf{M}'^{(3)}_{\ell' m'}(\mathbf{r}', k'_\mathrm{s})}{\sqrt{2}}, \tag{2}$$

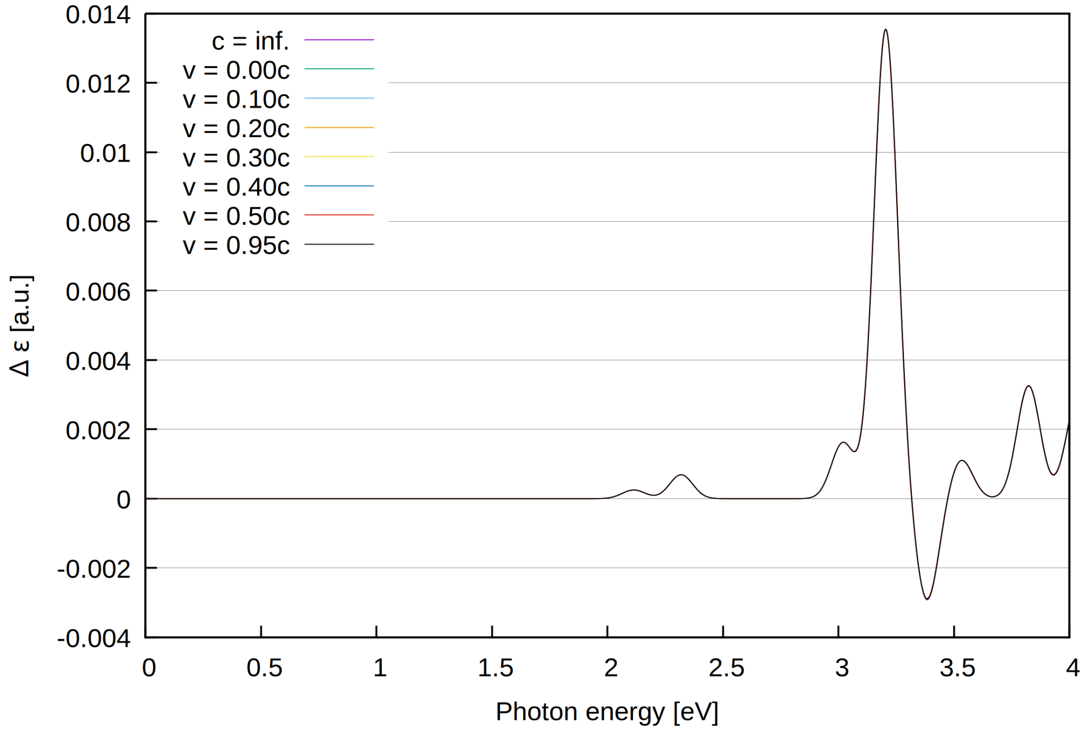

FIG. S1: Calculated circular dichroism spectra of chlorophyll a at velocities from $v = 0 - 0.95$ times the speed of light. The relativistic transformation outlined in Eq. 1 was used.

see equation (7) from[6]. $\mathbf{N}'^{(3)}_{\ell' m'}(\mathbf{r}', k'_\mathrm{s})$ corresponds to transverse magnetic (TM) modes and $\mathbf{M}'^{(3)}_{\ell' m'}(\mathbf{r}', k'_\mathrm{s})$ corresponds to transverse electric (TE) modes. With equations (11a) and (11b) from[6], we have

$$\mathbf{M}'^{(3)}_{\ell' m'}(\mathbf{r}', k'_\mathrm{s}) = N_{\ell' m'}\left(\mathrm{i}\pi'_{\ell' m'}(\theta'_\mathrm{s})\hat{\theta}'_\mathrm{s} - \tau'_{\ell' m'}(\theta'_\mathrm{s})\hat{\phi}'_\mathrm{s}\right)\mathrm{e}^{\mathrm{i}m'\phi'_\mathrm{s}} h^{(3)}_{\ell'}(k'_\mathrm{s}\mathbf{r}') = \mathbf{X}'_{\ell' m'}(\theta'_\mathrm{s}, \phi'_\mathrm{s}) h^{(3)}_{\ell}(k'_\mathrm{s}\mathbf{r})$$

$$\mathbf{N}'^{(3)}_{\ell' m'}(\mathbf{r}', k'_\mathrm{s}) = \left[\mathrm{i}\sqrt{\ell'(\ell'+1)}Y_{\ell', m'}(\theta'_\mathrm{s}, \phi'_\mathrm{s})\frac{h^{(3)}_{\ell'}(k'_\mathrm{s}\mathbf{r}')}{k'_\mathrm{s}r'}\hat{\mathbf{r}}' + \left(\frac{\mathrm{d}h^{(3)}_{\ell'}(k'_\mathrm{s}\mathbf{r}')}{\mathrm{d}(k'_\mathrm{s}\mathbf{r}')} + \frac{h^{(3)}_{\ell'}(k'_\mathrm{s}\mathbf{r}')}{k'_\mathrm{s}r'}\right)\hat{\mathbf{r}}' \times \mathbf{X}'_{\ell' m'}(\theta'_\mathrm{s}, \phi'_\mathrm{s})\right], \tag{3}$$

where $\pi'_{\ell' m'}(\theta'_\mathrm{s}) = \frac{m' P^{m'}_{\ell'}(\cos\theta'_\mathrm{s})}{\sin\theta'_\mathrm{s}}$ and $\tau'_{\ell' m'}(\theta'_\mathrm{s}) = \frac{\partial P^{m'}_{\ell'}(\cos\theta'_\mathrm{s})}{\partial\theta'_\mathrm{s}}$, see equations (13a) and (13b) from[6]. $P^{m'}_{\ell'}(\cos\theta'_\mathrm{s})$ are the asscoiated Legendre polynomials, see equation (B.2) from[6], $Y_{\ell', m'}(\theta'_\mathrm{s}, \phi'_\mathrm{s})$ are spherical harmonics, see equation (B.1) from[6], and $N_{\ell' m'} = \mathrm{i}\sqrt{\frac{2\ell'+1}{4\pi\ell'(\ell'+1)}\frac{(\ell'-m')!}{(\ell'+m')!}}$ is a normalisation factor, see equation (12) from[6]. $h^{(3)}_{\ell'}(k'_\mathrm{s}\mathbf{r})'$ are spherical Hankel functions of the first kind. In the limit of large distances, we derive

$$\mathbf{W}'^{(3)}_{\ell' m' \lambda'_\mathrm{s}}(r' \to \infty, k'_\mathrm{s}) = \left(\mathbf{Y}'_{\ell' m'}(\theta'_\mathrm{s}, \phi'_\mathrm{s})(-\mathrm{i})^{\ell'} + \lambda'_\mathrm{s}\mathbf{X}'_{\ell' m'}(\theta'_\mathrm{s}, \phi'_\mathrm{s})(-\mathrm{i})^{\ell'+1}\right) \cdot \frac{\mathrm{e}^{\mathrm{i}k'_\mathrm{s}r'}}{k'_\mathrm{s}r'} \tag{4}$$

$$= \mathbf{Q}'_{\ell' m' \lambda'_\mathrm{s}}(\theta'_\mathrm{s}, \phi'_\mathrm{s}) \cdot \frac{\mathrm{e}^{\mathrm{i}k'_\mathrm{s}r'}}{k'_\mathrm{s}r'}, \tag{5}$$

which is a vector in spherical coordinates. Here, $\mathbf{Y}'_{\ell' m'}(\theta'_\mathrm{s}, \phi'_\mathrm{s}) = \hat{\mathbf{r}}' \times \mathbf{X}'_{\ell' m'}(\theta'_\mathrm{s}, \phi'_\mathrm{s})$. Multiplying the coefficient vector $\mathbf{Q}'_{\ell' m' \lambda'_\mathrm{s}}(\theta'_\mathrm{s}, \phi'_\mathrm{s})$ with the scattered vector spherical wave expansion coefficients $B'_{\ell' m' \lambda'_\mathrm{s}}(k'_\mathrm{s})$, we have

$$\begin{pmatrix} E'_{\mathrm{s},\lambda'_\mathrm{s},r'_\mathrm{s}} \\ E'_{\mathrm{s},\lambda'_\mathrm{s},\theta'_\mathrm{s}} \\ E'_{\mathrm{s},\lambda'_\mathrm{s},\phi'_\mathrm{s}} \end{pmatrix} = \frac{1}{k'_\mathrm{s} r'} \sum_{\ell',m'} \mathbf{Q}'_{\ell' m' \lambda'_\mathrm{s}}(\theta'_\mathrm{s}, \phi'_\mathrm{s}) B'_{\ell' m' \lambda'_\mathrm{s}}(k'_\mathrm{s})\,. \tag{6}$$

Note that the $1/k'_\mathrm{s} r'$ component in equation (4) shows little variation for large r', which allows us to consider the expansion coefficients on the left hand side of equation (6) in the plane wave basis.

DIRECTION AVERAGED TCD SIGNAL FROM MOLECULES

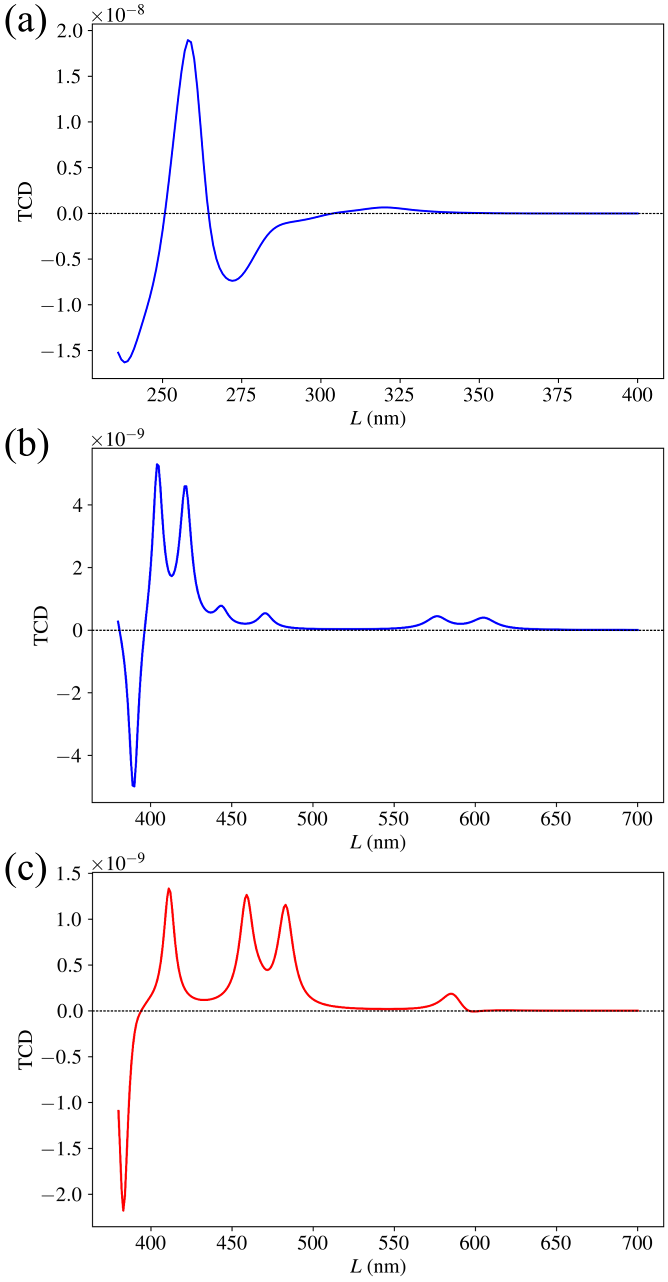

FIG. S2: Total TCD signal (with arbitrary scaling) scattered in all directions from the rotationally averaged (a) DNA molecular model; (b) Chlorophyll a and (c) Chlorophyll b molecules for speed ratio $\beta = 0$.

COMPUTATIONAL DATA FROM QUANTUM CHEMISTRY SIMULATIONS

All results of molecular quantum chemistry calculations can be found deposited to the NOMAD database for materials science under the following DOI: https://doi.org/10.17172/NOMAD/2024.02.07-1

AN EXAMPLE OF THE TURBOMOLE CONTROL FILE FOR ONE OF THE CALCULATIONS OF THE DYNAMIC POLARISABILITY TENSORS OF DNA MODEL

```
$cosmo
epsilon= 78.355
rsolv= 1.30
refind= 1.3333
$cosmo_atoms
# radii in Angstrom units
c 1,3,5-7,9-11,15,22-23,25-27,29-30,32,34,36,44-45,47-49,51,53-54,56,58,65-66
68-70,72,74-75,77,79,86-87,89-91,93,95-97,103,106-107,109-111,113,115-117,
123,126-127,129-131,133,135-137,145-146,148-150,152,154-155,157,159,167,169
171-173,175-177,181,187-191,195,197-198,200,202,208-209,211-213,218,220-222
225,228-229,231-233,238,240-242,245,248-249,251-253,258,260-261,263,265,
269-270,272-274,279,281-282,284,286,290-291,293-295,300,302-303,305,307,
312-313,315-317,322,324-326
radius= 2.0000
n 2,4,20,28,31,33,35,38,50,52,55,57,62,71,73,76,78,83,92,94,112,114,132,134,
142,151,153,156,158,161,166,168,170,194,196,199,201,203,217,219,237,239,257
259,262,264,266,278,280,283,285,287,299,301,304,306,308,321,323,328
radius= 1.8300
o 8,12,14,16,18-19,21,24,37,39,41-43,46,59,61,63-64,67,80,82,84-85,88,98,
100-102,104-105,108,118,120-122,124-125,128,138,140-141,143-144,147,160,
162-165,174,178,180,182,184-186,192-193,204-207,210,214,216,223-224,226-227
230,234,236,243-244,246-247,250,254,256,267-268,271,275,277,288-289,292,296
298,309-311,314,318,320,327,329-330
```

```
radius= 1.7200
h 331-530
radius= 1.3000
p 13,17,40,60,81,99,119,139,179,183,215,235,255,276,297,319
radius= 2.1060
$cosmo_out file=out.ccf
$cosmo_data file=cosmo_transfer.tmp
$title
$symmetry c1
$coord file=coord
$optimize
internal off
redundant off
cartesian on
$atoms
basis =def2-TZVP
jbas =def2-TZVP
$basis file=basis
$scfmo file=mos
$closed shells
a 1-1290 ( 2 )
$scfiterlimit 1000
$scfdamp start=0.300 step=0.050 min=0.100
$scfdump
$scfdiis
$maxcor 500 MiB per_core
$scforbitalshift automatic=.1
$energy file=energy
$grad file=gradient
$dft
functional pbe0
gridsize 3
```

$scfconv 7

$denconv 1.0d-7

$scftol 1.0d-15

$ricore 16000

$rij

$jbas file=auxbasis

$marij

$mgiao

$senex

gridsize 1

$esenex

$scfinstab dynpol nm

260

$damped_response 0.15 eV

$rpacor 140000 in_total

$escfiterlimit 300

$magnetic_response

$rundimensions

natoms=530

$last step mpshift

$end

CARTESIAN COORDINATES OF FINITE-SIZE DNA MODEL

C 6.24597 3.2973 10.01367

N 5.90497 2.1383 10.63567

C 4.61797 1.8333 10.93067

N 3.64397 2.6963 10.61667

C 3.92597 3.8523 10.01167

C 5.23497 4.1943 9.68567

C 6.98697 1.2153 11.02367

O 7.96497 2.0933 11.56767

C 9.23197 1.4823 11.35967

C 9.13297 0.8063 10.01767

C 7.63897 0.6553 9.77567

O 9.82997 -0.4417 10.04267

P 9.95797 -1.3387 8.73167

O 11.12897 -2.2157 8.87967

C 10.29297 2.5343 11.44967

O 9.96897 3.4953 10.44167

P 10.77297 4.8703 10.36667

O 12.21497 4.5653 10.33467

O 4.39397 0.7613 11.49667

N 2.92997 4.7483 9.74367

O 8.63997 -2.2137 8.78467

C 8.42297 -3.1257 9.86767

C 7.10197 -3.7907 9.61967

O 6.06197 -2.7987 9.61067

C 5.42497 -2.8497 8.33267

C 6.50197 -3.3827 7.40267

C 6.97297 -4.5217 8.29067

N 5.01397 -1.4697 7.98367

C 3.79297 -0.9257 8.23567

C 3.82997 0.3493 7.74967

N 5.02197 0.6433 7.19167

C 5.72597 -0.4877 7.34667

N 2.77697 -1.5717 8.83167

C 1.68097 -0.8207 8.93067

N 1.64197 0.4633 8.46667

C 2.66097 1.1533 7.86367

O 2.47797 2.3063 7.48267

N 0.53997 -1.3347 9.46767

O 5.99297 -5.5367 8.35267

P 6.06497 -6.7817 7.35967

O 6.54597 -6.3277 6.02467

O 9.91697 -0.4547 7.55467

O 4.51897 -7.1697 7.27567

C 3.72397 -7.1817 8.47667

C 2.38097 -6.6627 8.07167

O 2.55097 -5.3037 7.67567

C 1.55997 -5.0187 6.68367

C 1.66097 -6.2527 5.81067

C 1.72797 -7.3577 6.87867

N 2.09997 -3.8347 5.98167

C 3.36697 -3.6477 5.49667

N 3.57197 -2.4047 5.02767

C 2.38197 -1.7977 5.21267

C 1.45897 -2.6407 5.77567

N 0.17197 -2.3587 6.09867

C -0.11303 -1.0987 5.78367

N 0.67497 -0.1727 5.23267

C 1.93897 -0.4717 4.93367

O 0.47097 -7.9547 7.23467

P -0.42503 -8.7447 6.17767

O 0.42397 -9.2517 5.08267

N 2.75397 0.4713 4.38267

O 6.82497 -7.8597 8.00667

O -1.41203 -7.6397 5.59467

C -2.48903 -7.1537 6.40467

C -3.18003 -6.1477 5.54967

O -2.13903 -5.2907 5.07667

C -2.59203 -4.7767 3.83367

C -3.05103 -6.0587 3.17567

C -3.87403 -6.6807 4.30167

N -1.37303 -4.2247 3.19267

C -0.17603 -4.8517 2.98267

N 0.75797 -4.0367 2.47067

C 0.12497 -2.8497 2.36067

C -1.17503 -2.9277 2.77167

N -2.08103 -1.9267 2.81267

C -1.53803 -0.7887 2.38667

N -0.29203 -0.5597 1.96667

C 0.58197 -1.5637 1.92967

O -5.28403 -6.3817 4.29567

P -6.21003 -6.7007 3.02967

O -5.69103 -7.8567 2.25167

N 1.86997 -1.3327 1.54167

O -1.26303 -9.7117 6.91467

O -6.05203 -5.3637 2.19067

C -6.38403 -4.1157 2.80067

C -6.12903 -3.1227 1.71267

O -4.74803 -3.1707 1.39367

C -4.64703 -2.5417 0.13367

C -5.75903 -3.2237 -0.66233

C -6.83003 -3.4667 0.41067

N -3.27003 -2.8477 -0.32033

C -2.46903 -1.8277 -0.70933

N -1.17203 -2.1097 -1.03333

C -0.65803 -3.3787 -1.00333

C -1.51003 -4.4257 -0.65033

C -2.82603 -4.1347 -0.29133

O -8.04003 -2.7167 0.27667

P -8.90703 -2.7607 -1.06133

O -8.83303 -4.0827 -1.72533

O -2.90803 -0.6737 -0.74233

O 0.53897 -3.5287 -1.26633

C -1.02203 -5.8487 -0.64933

O -7.62303 -6.7907 3.45667

O -8.20503 -1.6327 -1.95833

C -8.14503 -0.2727 -1.46733

C -7.29603 0.4783 -2.44733

O -6.10303 -0.2927 -2.60133

C -5.55503 0.1043 -3.85133

C -6.77803 -0.0327 -4.74633

C -7.87303 0.5813 -3.85633

N -4.46803 -0.8747 -4.12633

C -3.20503 -0.4347 -4.39133

N -2.19703 -1.3477 -4.47333

C -2.41503 -2.6907 -4.33433

C -3.72203 -3.1397 -4.12033

C -4.74603 -2.2027 -3.98133

O -8.32404 1.9163 -4.18932

P -8.76004 2.3203 -5.67632

O -9.28104 1.1543 -6.41532

O -2.96703 0.7653 -4.49633

O -1.43303 -3.4337 -4.38233

C -4.03003 -4.6107 -3.98933

O -10.26603 -2.3067 -0.70533

O -7.36504 2.7963 -6.27432

C -6.65504 3.8763 -5.63633

C -5.39404 4.1023 -6.42833

O -4.65304 2.8763 -6.43733

C -4.05004 2.7323 -7.72833

C -5.16504 3.2143 -8.63233

C -5.57404 4.4893 -7.88733

N -3.76704 1.2713 -7.87033

C -2.47704 0.8523 -8.04533

N -2.18504 -0.4537 -8.01233

C -3.15004 -1.3557 -7.83933

C -4.48804 -0.9757 -7.70833

C -4.79204 0.3823 -7.71833

O -4.82104 5.6633 -8.21833

P -5.23604 6.5823 -9.46633

O -6.65904 6.3553 -9.83232

O -1.60004 1.6963 -8.21533

N -2.83004 -2.6757 -7.79633

O -9.64304 3.4933 -5.61632

O -4.33704 6.0143 -10.65633

C -2.90904 6.1893 -10.60533

C -2.31904 5.3393 -11.68733

O -2.48904 3.9733 -11.28933

C -2.86204 3.2773 -12.47233

C -3.87704 4.2113 -13.07133

C -2.96404 5.4273 -13.07333

N -3.40104 1.9623 -12.08533

C -4.67504 1.5643 -11.79533

N -4.75004 0.2583 -11.49833

C -3.47304 -0.1627 -11.60333

C -2.63504 0.8503 -11.94633

N -1.30904 0.8223 -12.15233

C -0.79304 -0.38569 -11.96733

N -1.56204 -1.4517 -11.60533

C -2.91504 -1.4587 -11.39633

O -1.98004 5.3453 -14.09933

N 0.54296 -0.5817 -12.12233

O -3.47804 -2.4987 -11.06433

O -4.79504 7.9663 -9.14332

O 10.26797 5.6613 9.22967

O 10.43197 5.5693 11.76767

N 0.39397 3.8773 7.51867

C -0.73103 3.1873 7.85367

N -0.65203 1.8883 8.14967

C -1.75703 1.1883 8.44367

N -2.96303 1.8053 8.44567

C -3.09803 3.1273 8.14967

C -1.95303 3.8583 7.85067

C -4.18403 0.9923 8.63767

O -4.81303 1.1513 7.37767

C -6.19103 0.8473 7.57567

C -6.52503 1.2943 8.99367

C -5.16803 1.6633 9.58367

O -7.25503 0.2903 9.72567

P -7.78203 0.5733 11.21367

O -8.90403 -0.3537 11.51067

C -6.96003 1.6013 6.54767

O -6.88203 2.9803 6.95368

P -7.69703 4.0723 6.13067

O -9.12203 3.6733 6.10168

O -1.73103 -0.0197 8.69367

O -6.52403 0.1733 12.10767

C -6.07503 -1.1917 12.18767

C -4.86903 -1.1937 13.09167

C -5.02403 -0.4837 14.43967

C -4.54003 0.9193 14.10467

C -3.30303 0.4753 13.37067

O -3.79803 -0.4907 12.43367

O -4.20303 -1.0937 15.40767

N -2.69203 1.6063 12.65767

C -3.25403 2.7943 12.27367

N -2.37003 3.6113 11.68067

C -1.21903 2.9023 11.69467

C -1.38903 1.6783 12.26567

N -0.47603 0.7013 12.46467

C 0.73197 1.0253 12.00567

N 0.97697 2.2323 11.42267

C 0.07897 3.2523 11.21667

N 1.77497 0.1633 12.15267

O 0.45597 4.3063 10.69967

O -8.05603 2.0233 11.42067

O -7.42103 5.4383 6.62568

O -7.09203 3.9063 4.66768

C -6.01903 4.7203 4.20167

C -5.63603 4.3093 2.79267

O -4.28803 3.8643 2.87867

C -4.10103 3.3973 4.20367

C -4.70503 4.5563 4.97467

C -5.71503 5.3973 1.77867

O -4.89603 6.4723 2.26767

P -4.77803 7.7823 1.37467

O -6.14604 8.2563 1.11267

N -2.63103 3.3193 4.31967

C -2.03403 2.1293 4.60067

N -0.67103 2.0783 4.61167

C 0.11097 3.1633 4.33967

C -0.51703 4.3753 4.04767

C -1.90603 4.4363 4.04067

O -2.70803 1.1153 4.80567

O 1.33397 3.0133 4.36667

C 0.27197 5.6073 3.72667

O -3.83603 8.7623 1.95567

O -4.18804 7.2013 0.00567

C -2.77603 7.0713 -0.21633

C -2.48204 6.4663 -1.58333

O -1.52404 5.4333 -1.35133

C -1.86503 4.8943 -0.08633

C -2.02403 6.1553 0.74467

C -1.89304 7.4203 -2.58233

O -0.65804 7.8773 -2.00033

P 0.31296 8.7943 -2.86533

O -0.49904 9.9453 -3.30333

N -0.68203 4.0993 0.31967

C -0.84903 2.8243 0.77067

N 0.25997 2.0743 1.02767

C 1.52397 2.5703 0.89167

C 1.68097 3.8963 0.47667

C 0.55297 4.6543 0.16267

O -1.98003 2.3503 0.91667

O 2.46397 1.8193 1.14367

C 3.05397 4.4883 0.34867

O 1.56096 9.0853 -2.13633

O 0.59895 7.8573 -4.12833

C 1.80995 7.1103 -4.27033

C 1.74395 6.2073 -5.49733

O 1.56496 4.8613 -5.03533

C 1.26996 4.9043 -3.64233

C 2.07696 6.0933 -3.16133

C 2.91196 6.2773 -6.43333

O 4.10496 6.1883 -5.64433

P 5.51196 6.3903 -6.35933

O 5.50496 7.7273 -6.99733

N 1.81296 3.6223 -3.13033

C 3.11996 3.2163 -3.05533

N 3.24796 1.9553 -2.60933

C 1.97496 1.5583 -2.40533

C 1.07596 2.5403 -2.71133

N -0.27204 2.4833 -2.62033

C -0.65504 1.2903 -2.17733

N 0.09796 0.2433 -1.84933

C 1.42397 0.3223 -1.95233

N 2.19196 -0.7567 -1.64233

O 6.66196 6.1073 -5.45933

O 5.44096 5.2593 -7.47433

C 6.05596 3.9923 -7.25233

C 5.80996 3.0373 -8.40133

O 4.62196 2.3033 -8.06933

C 4.20596 2.6423 -6.74033

C 5.46096 3.1943 -6.09433

C 6.92496 2.0823 -8.68533

O 7.28596 1.3563 -7.49533

P 8.61296 0.4873 -7.54634

O 9.70296 1.4323 -7.86034

N 3.78796 1.3443 -6.12733

C 4.55596 0.2693 -5.75233

N 3.82296 -0.7667 -5.30133

C 2.55096 -0.3267 -5.39733

C 2.49996 0.9553 -5.88333

N 1.40896 1.7213 -6.10233

C 0.30596 1.0503 -5.76733

N 0.19096 -0.1937 -5.29233

C 1.28596 -0.9337 -5.09933

N 1.15697 -2.2137 -4.64333

O 8.81296 -0.3607 -6.34734

O 8.30096 -0.4577 -8.78834

C 7.80996 -1.7917 -8.59234

C 7.44796 -2.4587 -9.90634

O 6.01396 -2.5257 -9.93334

C 5.47396 -1.7657 -8.83933

C 6.52896 -1.9217 -7.76434

C 7.88596 -3.8747 -10.09734

O 7.01496 -4.6337 -9.24834

P 7.01296 -6.2217 -9.32134

O 8.40496 -6.6637 -9.40934

N 4.22196 -2.4517 -8.41733

C 4.07196 -3.6887 -7.84633

N 2.78896 -4.0067 -7.60433

C 2.10896 -2.9187 -8.01733

C 2.94496 -1.9567 -8.50133

N 2.62096 -0.7337 -8.95733

C 1.31496 -0.4827 -8.91233

N 0.42096 -1.4017 -8.46733

C 0.70496 -2.6607 -8.00333

N 0.84296 0.7423 -9.26733

O -0.20904 -3.3727 -7.60533

O 6.19696 -6.7967 -8.23534

O 6.36796 -6.5317 -10.74334

C 4.96196 -6.6117 -10.95134

C 4.64496 -6.7927 -12.42133

O 3.30096 -6.3417 -12.50733

C 3.25396 -5.1487 -11.75733

C 4.22896 -5.3337 -10.61333

C 4.67196 -8.2197 -12.88133

O 3.66596 -8.8857 -12.09333

P 3.18696 -10.3707 -12.45433

O 4.37396 -11.2347 -12.61433

N 1.85396 -5.0547 -11.31333

C 1.21596 -3.8647 -11.44333

N -0.08204 -3.7677 -11.14933

C -0.75504 -4.8317 -10.71033

C -0.12704 -6.0617 -10.53733

C 1.21796 -6.1667 -10.86433

O 1.87096 -2.9047 -11.84233

N -2.08604 -4.7337 -10.43833

O 2.13596 -10.8107 -11.51833

O 2.54496 -10.1907 -13.89833

H 10.74311 6.48239 9.17926

H 11.25713 2.10906 11.26401

H 10.32857 2.97813 12.42263

H 9.49759 0.74999 12.0932

H 9.58753 1.36081 9.22345

H 7.33888 1.2108 8.9118

H 7.35948 -0.36166 9.59519

H 6.6407 0.42707 11.65903

H 2.02526 4.35455 10.03923

H 3.32883 5.5759 9.27795

H 5.45704 5.11999 9.19712

H 7.26769 3.51461 9.78181

H 10.70867 0.06952 7.52462

H 9.20361 -3.85697 9.89484

H 8.4228 -2.61218 10.80639

H 7.02196 -4.5111 10.40676

H 7.8646 -5.01763 7.96825

H 7.2728 -2.66444 7.21607

H 6.19067 -3.64618 6.41344

H 4.55032 -3.46427 8.28598

H 6.73409 -0.6098 7.00949

H 0.85489 1.12689 8.49915

H -0.19668 -0.61482 9.46218

H 0.71084 -2.30294 9.77461

H 7.46614 -6.09827 6.08083

H 3.64023 -8.17723 8.85983

H 4.16322 -6.58628 9.24961

H 1.74741 -6.82514 8.9185

H 2.27365 -8.19965 6.50685

H 2.54344 -6.23583 5.20581

H 0.86201 -6.36428 5.10774

H 0.55654 -4.82865 7.0029

H 4.11604 -4.41175 5.49157

H 3.68691 0.06619 4.22015

H 2.23198 1.35187 4.26847

H -1.11196 -0.78346 6.00199

H 1.01678 -9.91223 5.42145

H -3.15517 -7.94981 6.66424

H -2.14504 -6.7339 7.32681

H -3.94751 -5.70502 6.1496

H -3.89172 -7.74714 4.21621

H -2.22329 -6.67982 2.90372

H -3.58743 -5.91906 2.26042

H -3.34835 -4.01985 3.82583

H -0.00277 -5.8842 3.20365

H 2.40115 -2.21464 1.57216

H 1.97567 -0.34385 1.27354

H -2.19028 0.05949 2.37998

H -5.78994 -8.65187 2.76198

H -7.41023 -4.09785 3.10315

H -5.81859 -3.91955 3.68763

H -6.47781 -2.17826 2.07499

H -7.17752 -4.47612 0.33848

H -5.42119 -4.14544 -1.08796

H -6.10809 -2.64498 -1.49187

H -4.77262 -1.48096 0.07074

H -0.743 -1.21131 -1.29738

H 0.00456 -5.87391 -0.94996

H -1.11549 -6.25712 0.33523

H -1.60806 -6.42721 -1.33255

H -3.48628 -4.92221 0.00666

H -9.25382 -4.73571 -1.17851

H -9.12653 0.15111 -1.42322

H -7.73788 -0.22859 -0.4788

H -7.18417 1.47058 -2.06293

H -8.78904 0.04511 -3.99166

H -6.9856 -1.05788 -4.97179

H -6.67616 0.43657 -5.70252

H -5.1204 1.07684 -3.95217

H -1.3409 -0.81256 -4.67723

H -3.13196 -5.17654 -4.12426

H -4.4318 -4.80577 -3.017

H -4.74478 -4.89313 -4.73382

H -5.74312 -2.52329 -3.7624

H -10.11285 0.88827 -6.04141

H -7.25457 4.76256 -5.63905

H -6.42896 3.6362 -4.61841

H -4.91287 4.92688 -5.94517

H -6.56862 4.78165 -8.15237

H -5.96803 2.50901 -8.68409

H -4.88718 3.35805 -9.65557

H -3.13905 3.2578 -7.92541

H -1.81194 -2.78594 -7.90683

H -3.68573 -3.23209 -7.65802

H -5.25923 -1.70991 -7.60302

H -5.8014 0.72044 -7.60987

H -7.22041 6.68446 -9.14022

H -2.65968 7.2168 -10.76952

H -2.52582 5.90467 -9.64772

H -1.31416 5.69228 -11.78992

H -3.47849 6.34671 -13.2602

H -4.74044 4.34005 -12.45257

H -4.3236 3.92637 -14.00101

H -2.08113 3.05902 -13.17049

H -5.51952 2.22132 -11.80408

H -1.26187 -2.42169 -11.4324

H 0.76814 -1.57042 -11.94178

H 0.99074 0.30501 -12.39467

H 1.86068 -11.69181 -11.74276

H 5.63449 -8.65298 -12.70613

H 4.48272 -8.30688 -13.93085

H 5.36374 -6.27829 -13.02436

H 4.65682 -7.42503 -10.32661

H 3.70854 -5.42968 -9.68336

H 4.89043 -4.50023 -10.50071

H 3.52527 -4.25361 -12.27705

H -2.40213 -3.76915 -10.61335

H -2.43794 -5.64528 -10.11262

H -0.6675 -6.9053 -10.16165

H 1.73795 -7.0966 -10.76539

H 6.2095 -7.74457 -8.29766

H 8.90658 -3.9988 -9.80097

H 7.83134 -4.18462 -11.12002

H 7.93728 -1.87769 -10.65993

H 8.63017 -2.24496 -8.07589

H 6.46502 -2.87695 -7.28651

H 6.44881 -1.21845 -6.96188

H 5.25692 -0.74085 -9.05721

H 4.89293 -4.33535 -7.61664

H -0.60465 -1.32258 -8.4148

H -0.18252 0.75597 -9.17196

H 1.62516 1.33331 -9.58319

H 9.6201 -0.85328 -6.43891

H 7.77558 2.62894 -9.03538

H 6.60095 1.38527 -9.42969

H 5.72001 3.61775 -9.2957

H 7.07726 4.26713 -7.09013

H 6.11768 2.4109 -5.77824

H 5.28604 3.76665 -5.20736

H 3.40648 3.34814 -6.65357

H 5.62421 0.25618 -5.81216

H 2.08969 -2.64062 -4.55016

H 0.15977 -2.42845 -4.50057

H -0.61393 1.5813 -5.89674

H 7.4748 6.24206 -5.93222

H 2.89623 7.2041 -6.96784

H 2.87161 5.47945 -7.14516

H 0.9237 6.55933 -6.08738

H 2.55602 7.877 -4.29154

H 3.11799 5.85638 -3.09041

H 1.81081 6.43449 -2.18273

H 0.24564 5.006 -3.35024

H 3.94937 3.83696 -3.32321

H 3.18553 -0.52115 -1.77747

H 1.5868 -1.53338 -1.33996

H -1.71078 1.15323 -2.06996

H 2.12126 9.63286 -2.67368

H -2.55546 8.24422 -2.74742

H -1.73392 6.95163 -3.53098

H -3.40932 6.13713 -2.00369

H -2.44432 8.08081 -0.09082

H -1.07365 6.56768 1.01227

H -2.5266 5.99918 1.67631

H -2.73091 4.26921 -0.01984

H -0.08199 1.15882 1.35302

H 3.78442 3.7596 0.63212

H 3.22124 4.78515 -0.66563

H 3.13522 5.34225 0.98825

H 0.65566 5.65705 -0.19629

H -3.7919 9.52905 1.39652

H -6.72733 5.7254 1.66689

H -5.37752 5.0588 0.82138

H -6.32962 3.56577 2.45951

H -6.39361 5.71732 4.30438

H -4.09609 5.43422 4.9168

H -4.81374 4.39483 6.02682

H -4.51895 2.46322 4.51629

H -0.42428 1.10511 4.84166

H 1.31732 5.38464 3.77729

H 0.02585 5.94158 2.74047

H 0.03639 6.3758 4.43293

H -2.40249 5.35737 3.81692

H -7.92002 6.06638 6.11673

H -7.9784 1.27367 6.52583

H -6.56636 1.45124 5.5641

H -6.42812 -0.19094 7.47204

H -7.19569 2.12708 9.03344

H -5.02915 2.72421 9.5926

H -5.05313 1.34995 10.60029

H -3.9477 0.01037 8.99102

H 1.20905 3.24945 7.56723

H 0.14945 4.85859 7.32322

H -2.00841 4.90286 7.62543

H -4.06152 3.59268 8.1471

H -8.804 2.28237 10.8954

H -6.8454 -1.81071 12.59786

H -5.83298 -1.57662 11.21909

H -4.69717 -2.23216 13.28391

H -6.01306 -0.50871 14.84723

H -5.22319 1.45605 13.48009

H -4.41121 1.60279 14.91778

H -2.55036 0.08391 14.02275

H -4.28297 3.04336 12.42905

H 1.8687 2.58485 11.04661

H 2.62348 0.57772 11.74135

H 1.44471 -0.70136 12.60454

H -4.30708 -0.64571 16.23892

H -2.40238 5.40195 -14.9484

H 1.77488 -9.63792 -13.83584

H 9.50453 5.77208 11.80295

CARTESIAN COORDINATES OF CHLOROPHYLL *A*

C 14.8565723 -11.1775205 -1.2640108

C 15.7402192 -10.2063029 -0.7539117

C 13.4891158 -11.0492755 -1.3254457

N 15.5610303 -9.0162971 -0.1876489

C 17.1119029 -10.5620545 -0.8316675

C 17.8344436 -9.5057866 -0.2628608

C 16.8320195 -8.5502998 0.1361540

C 17.0145443 -7.3164558 0.7740326

C 16.0113261 -6.4224990 1.1533904

N 14.6600966 -6.6142595 0.9541806

C 16.2294636 -5.1534510 1.8236896

C 14.0249975 -5.5251354 1.4692981

C 14.9902183 -4.5943249 2.0213447

C 12.6303069 -5.3305778 1.4551392

C 11.6550290 -6.1623777 0.9188523

N 11.9034623 -7.3583539 0.2910924

C 10.7076738 -7.8611409 -0.1270538

C 9.6315113 -6.9638608 0.2458707
C 10.2198171 -5.8971528 0.8984012
C 10.5528635 -9.0727295 -0.8203887
C 11.5228086 -10.0059195 -1.1634292
N 12.8439177 -9.9203764 -0.8914695
C 12.5058017 -12.0852596 -1.8262509
C 11.2031257 -11.2630111 -1.9535615
C 9.6106496 -4.7238791 1.4961954
C 8.3873897 -4.2329860 1.2514107
C 8.1798788 -7.2050135 -0.0034226
C 14.6597129 -3.2829699 2.6576716
C 17.5646098 -4.5699405 2.1665507
C 18.1803940 -3.7817660 0.9993207
C 10.8446607 -10.9357358 -3.4075230
C 12.4148030 -13.2659359 -0.8412286
C 11.6573129 -14.4847112 -1.3937913
C 19.3106209 -9.3693179 -0.1081661
C 15.7222377 -12.3357182 -1.7269619
C 17.2111834 -11.8637531 -1.4460548
O 18.1854263 -12.5475919 -1.7250381
C 15.5671037 -12.6643826 -3.1950955
O 15.4367845 -11.8433462 -4.0798599
O 15.6093922 -13.9856805 -3.4079602
C 15.5127281 -14.4013842 -4.7932914
C 10.1706010 -14.2772659 -1.5413714
O 9.5593866 -14.2851085 -2.5936434
O 9.5855093 -14.0781463 -0.3503667
C 8.1434925 -13.7888041 -0.3792423
C 7.6736421 -13.5384403 1.0156785
C 7.9087869 -12.4152851 1.7068627
C 7.3278150 -12.2181761 3.0875547
C 8.7499355 -11.2939334 1.1725374

C 5.8772749 -11.6953562 3.0485298
C 5.7050132 -10.4016459 2.2547030
C 4.2986704 -9.7885961 2.3025239
C 4.2792065 -8.5036981 1.4607737
C 3.2312762 -10.7789496 1.8292384
C 3.0629933 -7.6057093 1.6891745
C 2.9523680 -6.4604861 0.6765261
C 4.1698781 -5.5290021 0.5864250
C 3.9437390 -4.4799588 -0.5117017
C 4.4786544 -4.8788626 1.9369508
C 5.2018314 -3.7144075 -0.9192653
C 4.9447988 -2.6872052 -2.0206886
C 6.2070722 -1.9980465 -2.5565335
C 5.8566181 -1.0892310 -3.7370260
C 6.9396004 -1.2050106 -1.4708854
Mg 13.7236586 -8.2202142 0.0513086
H 12.8167031 -12.4694429 -2.8028743
H 10.3607062 -11.7817419 -1.4922004
H 15.5690873 -13.2500951 -1.1486624
H 4.0823312 -9.5178381 3.3451562
H 5.0372454 -6.1311464 0.2878948
H 6.8846968 -2.7820836 -2.9198501
H 18.0325465 -7.0262064 1.0051512
H 12.2831853 -4.4086817 1.9059661
H 9.5431941 -9.3186963 -1.1249571
H 10.2286852 -4.1906988 2.2137140
H 8.0380569 -3.3475112 1.7688232
H 7.7138321 -4.6720523 0.5287284
H 7.8164693 -6.6345194 -0.8649047
H 7.5853713 -6.9000892 0.8617574
H 7.9768787 -8.2581454 -0.2002185
H 13.9037916 -3.3974095 3.4399333

H 14.2621503 -2.5739305 1.9243486
H 15.5451800 -2.8325401 3.1083568
H 18.2525160 -5.3656737 2.4673141
H 17.4603119 -3.9079902 3.0304605
H 19.1538861 -3.3728872 1.2812044
H 17.5293322 -2.9535003 0.7092612
H 18.3170995 -4.4241850 0.1259490
H 9.9595220 -10.2980818 -3.4562065
H 10.6389432 -11.8563683 -3.9578290
H 11.6715666 -10.4138884 -3.8961198
H 11.9541157 -12.9298574 0.0913385
H 13.4244131 -13.5979730 -0.5919658
H 11.8037976 -15.3216896 -0.7062122
H 12.0529702 -14.7696716 -2.3695001
H 19.5740012 -8.5173829 0.5192405
H 19.7891370 -9.2317668 -1.0831637
H 19.7393444 -10.2713010 0.3356238
H 14.5719441 -14.0528152 -5.2178475
H 15.5489689 -15.4865069 -4.7662792
H 16.3505406 -13.9958628 -5.3589695
H 7.6426453 -14.6454327 -0.8281930
H 8.0042577 -12.9265799 -1.0343932
H 7.0978085 -14.3333791 1.4812958
H 7.9511705 -11.5120683 3.6459040
H 7.3379550 -13.1657739 3.6330123
H 9.7501521 -11.3330618 1.6174289
H 8.3265841 -10.3234569 1.4359908
H 8.8687966 -11.3413629 0.0910183
H 5.5364856 -11.5424261 4.0790537
H 5.2483986 -12.4790578 2.6167063
H 5.9639464 -10.5856037 1.2044438
H 6.4168677 -9.6537823 2.6249123

H 4.3356854 -8.7804293 0.3992886

H 5.1897714 -7.9344226 1.6766325

H 2.2421208 -10.3160181 1.8029273

H 3.1692204 -11.6499286 2.4851662

H 3.4649864 -11.1324912 0.8189781

H 3.0993446 -7.2071068 2.7090924

H 2.1461566 -8.2004959 1.6273926

H 2.0684562 -5.8551925 0.9148049

H 2.7766729 -6.8901351 -0.3173372

H 3.1743675 -3.7717159 -0.1770903

H 3.5380079 -4.9820560 -1.3984738

H 5.3236639 -4.1908835 1.8679626

H 4.7258536 -5.6264520 2.6945151

H 3.6111057 -4.3125923 2.2924860

H 5.9555518 -4.4316402 -1.2690159

H 5.6279429 -3.2135686 -0.0458784

H 4.2518825 -1.9207224 -1.6488436

H 4.4375969 -3.1839142 -2.8569810

H 5.3577720 -1.6506578 -4.5323145

H 6.7529348 -0.6246998 -4.1579785

H 5.1820360 -0.2879799 -3.4166079

H 7.8179339 -0.7017547 -1.8849316

H 7.2772963 -1.8484302 -0.6553318

H 6.2812195 -0.4392019 -1.0464309

CARTESIAN COORDINATES OF CHLOROPHYLL *B*

C 15.8629869 -11.3483199 0.2319277

C 17.1116472 -10.6976468 0.2663895

C 14.7122897 -10.8172581 -0.2992127

N 17.5301091 -9.4995407 -0.1253956

C 18.1509517 -11.4592046 0.8669648
C 19.2983792 -10.6654516 0.8460563
C 18.8778277 -9.4331772 0.2150838
C 19.6551409 -8.3054442 -0.0350480
C 19.2410269 -7.1080139 -0.6441819
N 17.9463993 -6.8847928 -1.1014939
C 20.0531016 -5.9456650 -0.8908087
C 17.9019327 -5.6394431 -1.6244513
C 19.2053552 -5.0200739 -1.5092908
C 16.7541263 -5.0415616 -2.2002709
C 15.4875951 -5.5786243 -2.3441133
N 15.1110674 -6.8354299 -1.9360447
C 13.8015834 -6.9997647 -2.2667642
C 13.2949794 -5.8030861 -2.9175942
C 14.3465811 -4.9139782 -2.9742546
C 13.0558039 -8.1638373 -2.0253030
C 13.4670811 -9.3383777 -1.4066637
N 14.6878441 -9.5749589 -0.8791326
C 13.3428617 -11.4592063 -0.3098585
C 12.5701132 -10.5581241 -1.3002579
C 14.3928403 -3.5656167 -3.5098215
C 13.5619594 -3.0311082 -4.4158131
C 11.8913748 -5.6088903 -3.3834055
C 19.5229698 -3.6889549 -1.9641388
C 21.4986749 -5.6597982 -0.5985523
C 22.3671174 -6.7095038 0.0905164
C 12.3680911 -11.2149147 -2.6717837
C 12.7355593 -11.4859046 1.1070708
C 11.5467146 -12.4503745 1.2497907
C 20.6672746 -10.9959658 1.3314346
C 16.0689484 -12.7030453 0.8837244
C 17.6102357 -12.7348807 1.2740452

O 18.1398166 -13.6946830 1.8117031
C 15.7721065 -13.8711365 -0.0412171
O 15.9068954 -13.8054682 -1.2452673
O 15.3705036 -15.0349190 0.4913866
C 15.1670624 -15.1862387 1.9196457
C 10.3549500 -12.0857444 0.3993266
O 9.9543916 -12.7274720 -0.5515367
O 9.7903439 -10.9391955 0.8139692
C 8.6271560 -10.4667545 0.0483125
C 7.3686314 -11.0872531 0.5483809
C 6.5118464 -11.8307624 -0.1633530
C 5.2463500 -12.3487309 0.4721158
C 6.7047620 -12.1896698 -1.6102804
C 3.9660515 -11.8476275 -0.2190821
C 3.8061514 -10.3303848 -0.1428245
C 2.5542212 -9.7839163 -0.8435458
C 2.3309036 -8.3178607 -0.4453219
C 2.6506487 -9.9521160 -2.3625723
C 1.0203793 -7.7114270 -0.9477033
C 0.7958951 -6.2637748 -0.4962654
C 0.7304354 -6.0306593 1.0213212
C 0.5388356 -4.5330070 1.3000622
C -0.3672833 -6.8745537 1.6734989
C 0.6859269 -4.1272000 2.7666927
C 0.5523102 -2.6180349 2.9710445
C 0.7653337 -2.1420846 4.4145149
C 0.7920705 -0.6127381 4.4721984
C -0.2997851 -2.6918407 5.3672014
Mg 16.3073866 -8.1781706 -1.0155037
O 20.6216275 -3.1395974 -1.8778211
H 13.4063926 -12.4848138 -0.6853938
H 11.5987351 -10.2647418 -0.8979171

H 15.5130026 -12.8038995 1.8178533
H 1.6889069 -10.3600907 -0.4868276
H 1.6929739 -6.3242572 1.4588802
H 1.7443160 -2.5120895 4.7468851
H 20.6850212 -8.3603216 0.2767792
H 16.8872047 -4.0389370 -2.5844850
H 12.0309237 -8.1557728 -2.3761044
H 15.1968205 -2.9382555 -3.1338775
H 13.6901082 -2.0046665 -4.7385230
H 12.7576080 -3.5910194 -4.8751437
H 11.7913275 -5.8164017 -4.4541462
H 11.5709602 -4.5767023 -3.2213578
H 11.2021875 -6.2684147 -2.8542099
H 18.6872913 -3.1350552 -2.4273558
H 21.5262869 -4.7355334 -0.0114535
H 21.9658694 -5.3816162 -1.5495757
H 23.3731260 -6.3021441 0.2118201
H 22.4543907 -7.6251423 -0.4989344
H 21.9952089 -6.9636589 1.0858596
H 11.8832802 -10.5248995 -3.3654606
H 11.7413969 -12.1026934 -2.5696289
H 13.3299055 -11.5116892 -3.0978611
H 12.4371846 -10.4741969 1.3936448
H 13.4968417 -11.8016092 1.8237496
H 11.2271653 -12.4578655 2.2946514
H 11.8477782 -13.4613009 0.9716312
H 21.2487542 -10.0980473 1.5445034
H 21.2108473 -11.5735827 0.5760710
H 20.6205637 -11.6050028 2.2357613
H 14.3144871 -14.5874299 2.2425938
H 16.0697129 -14.9132357 2.4626493
H 14.9473857 -16.2413998 2.0567081

H 8.8100270 -10.6633601 -1.0054390

H 8.6393825 -9.3917962 0.2295165

H 7.1321889 -10.8750386 1.5886966

H 5.2256910 -12.0651252 1.5287290

H 5.2571528 -13.4448995 0.4257106

H 6.0665665 -11.5722372 -2.2513432

H 6.4110594 -13.2300820 -1.7790018

H 7.7370466 -12.0674820 -1.9340753

H 3.1037771 -12.3269069 0.2564868

H 3.9687396 -12.1789519 -1.2614181

H 4.6920596 -9.8448069 -0.5710494

H 3.7778292 -10.0372083 0.9138513

H 3.1737071 -7.7154793 -0.8107321

H 2.3604606 -8.2572103 0.6479565

H 1.7414936 -9.6137501 -2.8641438

H 2.8095287 -10.9963985 -2.6410665

H 3.4903770 -9.3675887 -2.7543168

H 0.1852261 -8.3396418 -0.6174964

H 1.0008883 -7.7307799 -2.0416508

H -0.1389060 -5.8972512 -0.9394768

H 1.5983806 -5.6379782 -0.9060953

H -0.4511767 -4.2278157 0.9356201

H 1.2719883 -3.9724843 0.7067819

H -0.4187494 -6.7008074 2.7505735

H -0.1950188 -7.9426461 1.5213045

H -1.3443185 -6.6269020 1.2440107

H 1.6666884 -4.4551176 3.1342590

H -0.0628431 -4.6470201 3.3708318

H -0.4410140 -2.2919861 2.6343688

H 1.2792851 -2.1099935 2.3253346

H 1.5720853 -0.2070506 3.8214067

H 0.9788361 -0.2587838 5.4902310

H -0.1681697 -0.2001409 4.1442221

H -0.1345081 -2.3278619 6.3853217

H -0.2954581 -3.7838160 5.3977100

H -1.2972707 -2.3668716 5.0515579

REFERENCES

[1]D. Peng, N. Middendorf, F. Weigend, and M. Reiher, "An efficient implementation of two-component relativistic exact-decoupling methods for large molecules," J. Chem. Phys. **138**, 184105 (2013).

[2]C. Holzer and Y. J. Franzke, "Beyond electrons: Correlation and self-energy in multicomponent density functional theory." ChemRxiv (2024).

[3]J. P. Perdew, K. Burke, and M. Ernzerhof, "Generalized gradient approximation made simple," Phys. Rev. Lett. **77**, 3865–3868 (1996).

[4]C. Adamo and V. Barone, "Toward reliable density functional methods without adjustable parameters: The PBE0 model," J. Chem. Phys. **110**, 6158–6170 (1999).

[5]P. Pollak and F. Weigend, "Segmented contracted error-consistent basis sets of double- and triple-ζ valence quality for one- and two-component relativistic all-electron calculations," J. Chem. Theory Comput. **13**, 3696–3705 (2017).

[6]D. Beutel, I. Fernandez-Corbaton, and C. Rockstuhl, "treams–a t-matrix-based scattering code for nanophotonics," Computer Physics Communications , 109076 (2024).
